# Effects of autochthonous strains mixture on gut microbiota and metabolic profile in cobia (*Rachycentron* *canadum*)

**DOI:** 10.1038/s41598-022-19663-x

**Published:** 2022-10-18

**Authors:** Eric Amenyogbe, Jun Luo, Wei-jie Fu, Emmanuel Delwin Abarike, Zhong-liang Wang, Jian-sheng Huang, Christian Larbi Ayisi, Gang Chen

**Affiliations:** 1 Fishery College, Guangdong Ocean University, Zhanjiang, 524025 China; 2 Guangdong Provincial Key Laboratory of Pathogenic Biology and Epidemiology for Aquatic Economic Animals, Zhanjiang, 524088 China; 3 Department of Fisheries and Aquatic Resources Management, University for Development Studies, Nyankpala-Campus, Post Box TL 1882, Nyankpala, Ghana; 4 Department of Water Resources and Sustainable Development, University of Environment and Sustainable Development, PMB SOMANYA, Somanya, Ghana

**Keywords:** Biochemistry, Genetics, Immunology, Microbiology, Physiology

## Abstract

The fish immune system is a topic or subject that offers a unique understanding of defensive system evolution in vertebrate heredity. While gut microbiota plays several roles in fish: well-being, promoting health and growth, resistance to bacterial invasion, regulation of energy absorption, and lipid metabolism. However, studies on fish gut microbiota face practical challenges due to the large number of fish varieties, fluctuating environmental conditions, and differences in feeding habits. This study was carried out to evaluate the impacts of supplemented three autochthonous strains, *Bacillus* sp. RCS1, *Pantoea* *agglomerans* RCS2, and *Bacillus* *cereus* RCS3 mixture diet on cobia fish (*Rachycentron* *canadum*). Also, chromatography, mass spectrometry and high throughput sequencing were combined to explore composition and metabolite profile of gut microbiota in juvenile cobia fed with supplemented diet. In the trial group, juvenile cobia received diets supplemented with 1 × 10^12^ CFU mL^−1^ autochthonous strains for ten weeks and a control diet without supplementation. Juvenile cobia receiving diets supplementation exhibited significantly improved growth than those without additives (control). Haematological indices, such as red blood cells, white blood cells, corpuscular haemoglobin concentration, mean corpuscular volume, haemoglobin, and mean corpuscular haemoglobin, were higher in the supplemented group. Similarly, digestive enzymes (trypsin, lipase, amylase, pepsin and cellulose, activities) activities were higher in supplemented diet with an indigenous isolates mixture. Serum biochemical parameters albumin, globulin, and total protein were significantly higher, while triglyceride, alanine aminotransferase, aspartate aminotransferase, alkaline phosphatase, and cholesterol showed no significant difference. On the other hand, glucose was significantly (*P* < 0.05) higher in the group without supplementation. On gene expression in the midgut, Immunoglobulin, Colony-stimulating factor receptor 1, major histocompatibility complex 1 were up-regulated by native isolates while T cell receptor beta, and Major histocompatibility complex 2 showed no significant difference. Gut bacterial composition was altered in fish receiving supplemented diet with autochthonous strains. Metabolomics also revealed that some metabolic pathways were considerably enriched in fish fed with supplemented diet; pathway analysis based on Kyoto Encyclopedia of Genes and Genomes (KEGG) enrichment revealed that differentially expressed metabolites were involved in galactose metabolism, tryptophan metabolism, carbohydrate digestion and absorption, purine metabolism, and ABC transporters. Functional analysis of bacterial community showed that differences in enriched metabolic pathways generally comprised carbohydrate and its metabolites, nucleotide and its metabolites, amino acid and its metabolites, heterocyclic compounds, and tryptamines, cholines, pigments. The current investigation results showed that autochthonous strains mixture has significantly enhanced the growth, survival, and innate and adaptive immunities of juvenile cobia.

## Introduction

Cobia fish (*Rachycentron* *canadum*) has become a globally appreciated and well-known cultured aquaculture species cultivated in several aquaculture systems. In the southern coasts of China, cobia forms an essential part of the marine cage culture industry^1^. Even though cobia is comparatively more resistant to infections and diseases than other aquaculture cultured species, serious infection-associated problems have gradually developed, including several bacteriological infections, comprising furunculosis and mycobacteriosis vibriosis, and streptococcosis as well as *Photobacterium* sp., a disease-causing bacteriological species that has been recognized as momentous developing trouble faced by cobia culture as a result of the increased intensive culture^2^.

The utilization of antibiotic treatment has been usually the most common approach to face these bacteriological infections and disease problems^3^. Nevertheless, there is a general alarm that antibiotic utilization in aquaculture has resulted in a rise and choice of resistant microbes^4,5^. A substitute for controlling and averting pathogenic microorganisms is the utilization of probiotics^6^. A suitable description of ‘probiotic’ in aquaculture as specified by the Food and Agriculture Organization (FAO) is live microorganisms with health benefits when administered in sufficient amounts^7^. While Verschuere et al.^8^ described probiotic as any live bacterial adjunct that has a positive influence on the organism via alterations in the host-associated or ambient bacterial community, thru an enhancement in the utilize of diet or its nutritious value, or via improving the organism reaction to disease or through enhancing its environmental quality.

Additionally, probiotics yield digestive enzymes that, via stimulating food absorption, create more significant utilization of consumed diet and ultimately lead to better growth^9^. Thus, probiotics could be a suitable substitute for growth stimulators of antibiotics, the ingestion that is being restricted^4,10,11^. In addition to understanding the physiology of a fish species, it is essential to identify its haematological parameters. Besides allowing for species identification, it can also provide useful information regarding the disease status of fish and the health of the fish^12^.

The gut microbiota composition is impacted via numerous factors, comprising microorganisms existing in the aquaculture environment and vice versa. Bacterial communities in the digestive tract are vital components of the host mucosal barrier defences^13^. Competition for adhesion sites and nutrients may limit or decrease the abundance of pathogens in the gut. In modern aquaculture practices, several efforts have been made to isolate potential probiotics species from guts for the cultured species, and practical effects were detected in immune stimulation, plummeting the occurrence of diseases and growth promotion^14–16^; though, undesirable influences of some microbes were also documented^17^. The utilization of probiotics of multi-species as feed additives is perhaps more effective than monospecies in terms of higher adhesion and multiplicity of antimicrobial compounds on the intestinal mucus^18,19^.

It is believed that T and B lymphocytes (T and B Cells) play a role in the acquired or antigen-specific immune response, as they are the only cells within the organism capable of recognizing and responding to each antigenic epitope^20^. In addition to producing antibodies, B cells can transform into plasmocytes. Therefore, humoral immunity depends on B cells, and cellular immunity depends on T cells^20^. Upon infection resolution, they sustain an improved resistance against the stimulating agent via memory cells tenacity^21^. The memory and specificity of B and T cells result from a rare cell being explicit for stimulating antigen production and performing the adaptive reaction, as articulated in clonal immunity theory^21^. A poorly understood source and development of this unique protective system persist during vertebrate evolution. Nonetheless, it is imperative to recognize that the lymphoid tissues organization, where lymphocytes advance, encounter antigens, and are triggered, differs significantly between mammals and fish^22^.

On the surface cells, major histocompatibility complex (MHC) molecules are expressed and identified as usual and "self" to natural killer (NK) cells. They (MHC) molecules play an essential role in foreign antigens presentation, a critical step in the T cells stimulation and hence a critical mechanism of an adaptive immune system. MHC is a group of genes coding for MHC molecules established on all nucleated cells surface of the body^23^. There are two classes of MHC molecules in adaptive immunity, namely MHC I and MHC II. MHC I molecules are found on all nucleated cells and release normal self-antigens and nonself or abnormal pathogens to the effector T cells that are intricate in cellular immunity. In distinction, only on dendritic cells**,** B cells, and macrophages that those of MHC II molecules can be found and release abnormal pathogen antigens for the preliminary stimulation of T cells^24^. In vertebrates comprising teleost fishes, the major histocompatibility complex (MHC) is an essential component of the immune response and is accountable for the foreign antigens presentation^25^.

Regarding the microbes utilized in the present study, one of the most considered probiotics is *Bacillus* sp., in fish species and described as having several valuable properties. Comprising immunostimulation as well as pathogens resistance improvement; they also have been reported to have the capacity to inhabit the digestive tract and modify the usual equilibrium of the gut microbiota when utilized as an additive in fish diets^26^. While the strains of *Pantoea* genus, comprising *Pantoea* *agglomerans* BSL 2, are commercially utilized as biological control agents, bio-remediation control, drug, and their competence of numerous regulatory functions of animal pathogen interactions^2,27–29^). Creating a better understanding of autochthonous strains (*Bacillus* sp. RCS1, *P.* *agglomerans* RCS2, and *Bacillus* *cereus* RCS3) mixture of probiotics’ effects on growth, haematology, digestive enzyme activities, antioxidants activities, disease resistance on the expression of the immune-related genes, on gut microbiota and its associated metabolic profiles in cobia fish (*R.* *canadum*), their applications, and their mechanisms of action on cobia after ten weeks of feeding (culture) was the purpose of this study. This is the first study that combines autochthonous strains *Bacillus* sp. RCS1, *P.* *agglomerans* RCS2, and *B.* cereus RCS3 as supplements in the cobia diet to the best of our knowledge.

## Results

The feeding approach did not result in any fish mortality throughout the trial. The results indicated efficiency in stimulating improved fish growth, exhibited higher weight gain, final weight, and reduced feed conversion ratio (FCR) (*P* < 0.05) (Table 1). The dietary autochthonous strains mixture additive at 1 × 10^12^ CFU/mL largely stimulated the growth performance of juvenile cobia (Table 1).

**Table 1:** The growth performance of juvenile cobia fed autochthonous strains supplemented diets and control diets.

Growth parameters	CT	MIX
IW (g)	183.87 ± 0.95^a^	183.77 ± 0.64^a^
FW (g)	4432.76 ± 46.1281^a^	5588.54 ± 51.24^b^
WG (g)	2310.85 ± 35.06^a^	3040.57 ± 51.24^b^
SGR (%/day)	4.55 ± 0.02^a^	4.88 ± 0.02^b^
FCR	1.23 ± 0.013^b^	0.98 ± 0.01^a^
Survival rate (%)	100	100

Different superscript letters indicate that the corresponding values are significantly different (*P* < 0.05) from the control. *FCR* Food conversion ratio, *WG* weight gain, *SGR* specific growth rate, *IW* Initial weight, *WG* weight gain, *SGR* specific growth rate, *CT* Control, *MIX* autochthonous strains mixture concentration at 1 × 10^12^ CFU/mL. Results are articulated as mean ± standard error (M ± SE).

**Figure 1:**
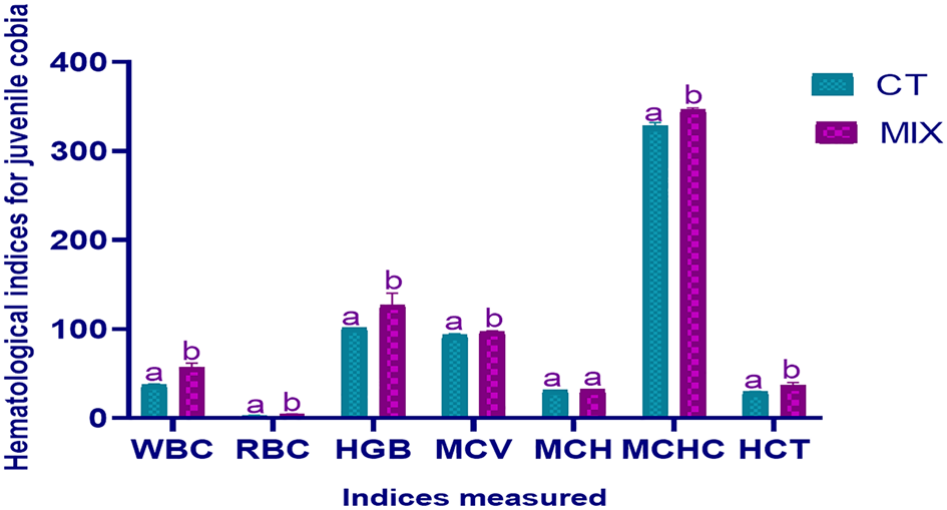
Hematological indices for juvenile cobia fed diets containing varying amounts of autochthonous strains on cobia fish after ten weeks. The number of fish per treatment is six. By independent T-test, different letters (a and b) indicate significant differences (*P* < 0.05). *RBCs* red blood cells (× 10^12^/L), *HGB* Hemoglobin (g/L), *WBCs* white blood cells (× 10^9^/L), *MCH* mean corpuscular haemoglobin (pg), *HCT* haematocrit (%), *MCHC* mean corpuscular haemoglobin concentration (g/L), *MCV* mean corpuscular volume (fL), *MIX* autochthonous strains mixture concentration at 1 × 10^12^, *CT* Control. Results are articulated as mean ± standard error (M ± SE).

After feeding cobia fish with the autochthonous strains supplement for 70 days, the result of the study shows that there were significant differences (*P* < 0.05) in the number of red blood cells and white blood cells, the concentration of corpuscular haemoglobin, the mean corpuscular volume, and the haemoglobin content of the corpuscles in the juvenile cobia fed with supplementary diet (Fig. 1) while no noticeable change was observed in MCVs. The distinction counts of all indices verified showed that the focal impact of supplementation was substantial (*P* < 0.05) (Fig. 1), excluding MCVs.

**Figure 2:**
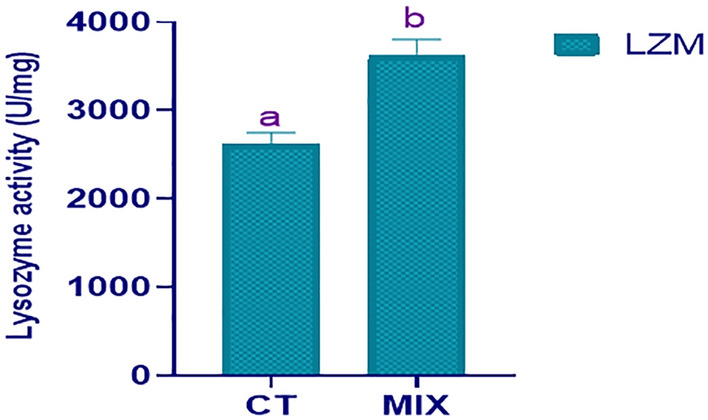
Influences of dietary supplemented autochthonous strains mixture on juvenile cobia's blood lysozyme activity (U mg^−1^). Results are articulated as mean ± standard error (M ± SE). According to independent T-test, different letters (a and b) indicate significant differences (*P* < 0.05). *MIX* autochthonous strains mixture concentration at 1 × 10^12^, *CT* Control. n = 24.

After 70 days trial, the globulin, total protein, and albumin in the blood were significantly different (*P* < 0.05) between the control and treatment group as the present results indicated. There were no significant differences in ALT, AST, TG, CHO, and GLU levels between the control and treatment groups (*P* > 0.05) (Table 2). The lysozyme activity of blood in the mixture treatment group significantly differs as compared to the control group (Fig. 2).

**Table 2:** The blood biochemistry of juvenile cobia fed autochthonous strain formulated diets after 10 weeks.

Blood serum indices	CT	MIX
ALT (U/L)	3.621 ± 1.403^a^	2.724 ± 0. 703^a^
AST (U/L)	9.821 ± 1.930^a^	15.826 ± 1.992^a^
TG (mmol/L)	1.498 ± 0.308^a^	1.415 ± 0.105^a^
CHO (mmol/L)	3.782 ± 0.118^a^	4.95 ± 0.213^a^
GLU (mmol/L)	7.158 ± 0.406^a^	5.453 ± 0.227^a^
TP (g/L)	30.790 ± 0.866^a^	39.327 ± 0.888^b^
ALB (g/L)	9.720 ± 0.2562^a^	13.722 ± 1.246^b^
GLOB (g/L)	21.071 ± 0.690^a^	28.053 ± 0.647^b^

The number of fish per treatment is six. The rows' letters represent significant differences (*P* < 0.05). Results are articulated as mean ± standard error (M ± SE). *ALT* alanine aminotransferase, *AST* aspartate aminotransferase, *TG* Triglycerides, *CHO* Cholesterol, *GLU* Glucose, *TP* Total protein, *ALB* Albumin, *GLOB* Globulins, *CT* Control, *MIX* autochthonous strains mixture concentration at 1 × 10^12^.

In this study, we also examine the effect of autochthonous strains mixture supplemented diets on the antioxidant enzymes activities such as superoxide dismutase (SOD), catalase (CAT), and glutathione peroxidase (GPx) in both liver and head kidney of juvenile cobia. These parameters were significantly higher in the treatment group compared to the control group. No substantial change was detected concerning the malondialdehyde (MDA) in the head kidney and liver. There were no observations of significant differences between the trial groups (Table 3). The autochthonous strains mixture supplemented diets also enhanced the digestive enzymes indices comprising cellulose, pepsin, amylase, lipase, and trypsin (Table 4).

**Table 3:** The antioxidant enzyme activities of juvenile cobia fed autochthonous strain formulated diets after 10 weeks.

Names	CT	MIX
**Liver**		
Superoxide dismutase (U/mgprot)	626.534 ± 35.46^a^	826.841 ± 37.179^b^
Catalase (U/mgprot)	27.806 ± 2.291^a^	45.373 ± 0.069^b^
Glutathione peroxidase (U/mgprot)	3.787 ± 0.235^a^	5.191 ± 0.332^b^
Malondialdehyde (nmol/mgprot)	0.652 ± 0.112^a^	0.542 ± 0.029^a^
**Head kidney**		
Superoxide dismutase (U/mgprot)	626.676 ± 197.024^a^	899.951 ± 46.139^b^
Catalase (U/mgprot)	4.014 ± 0.512^a^	6.138 ± 0.458^b^
Glutathione peroxidase (U/mgprot)	51.662 ± 10.345^a^	74.333 ± 3.073^b^
Malondialdehyde (nmol/mgprot)	103.819 ± 17.664^a^	105.381 ± 23.194^a^

Different letters on the rows indicate significant differences (*P* < 0.05). Results are articulated as mean ± standard error (M ± SE). *CT* Control, *MIX* autochthonous strains mixture concentration at 1 × 10^12^ CFU/mL.

**Table 4:** Digestive enzyme activities of juvenile cobia fed with autochthonous strains supplemented diets and diet without supplementation for 10 weeks.

Names	CT	MIX
Trypsin (U/mgprot)	881.280 ± 113.143^a^	1799.309 ± 63.446^b^
Amylase (U/mgprot)	0.304 ± 0.068^a^	0.514 ± 0.0501^b^
Cellulase (U/mgprot)	22.015 ± 0.495^a^	30.812 ± 1.484^b^
Pepsin (U/mgprot)	2.764 ± 0.451^b^	3.877 ± 0.368^b^
Lipase (U/gprot)	4.786 ± 0.084^a^	6.964 ± 0.356^b^

Different letters on the rows indicate significant differences (*P* < 0.05). Results are articulated as mean ± standard error (M ± SE). *CT* Control, *MIX* autochthonous strains mixture concentration at 1 × 10^12^ CFU/mL.

**Figure 3:**
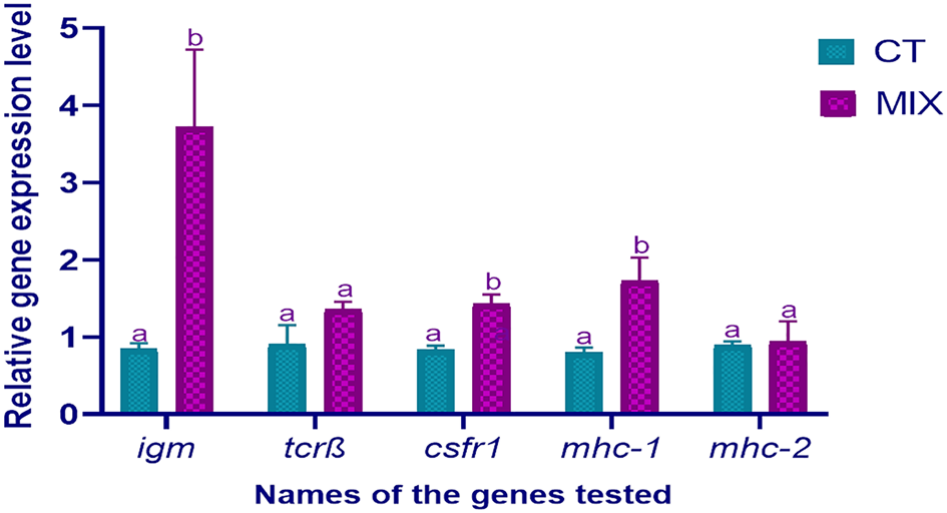
Relative expression of *igm*, *tcrβ*, *csfr1*, *mhc-1*, and *mhc-2* gene in the gut tissues of cobia. Letters (a and b) show significant differences (*P* < 0.05). *MIX* autochthonous strains mixture concentration at 1 × 10^12^, *CT* Control.

The expression of T cell receptor beta (Tcrβ), and major histocompatibility complex 2 (MHC-2) exhibited no significant difference (*P* > 0.05) among the groups tested. There were significant differences (*P* < 0.05) among groups in the case of Immunoglobulin (IgM), colony-stimulating factor receptor (Csfr1), and major histocompatibility complex 1 (MHC-1) in the present study (Fig. 3). However, the Tcrβ mRNA expression exhibited variations in the control and trial groups (Fig. 3).

**Figure 4:**
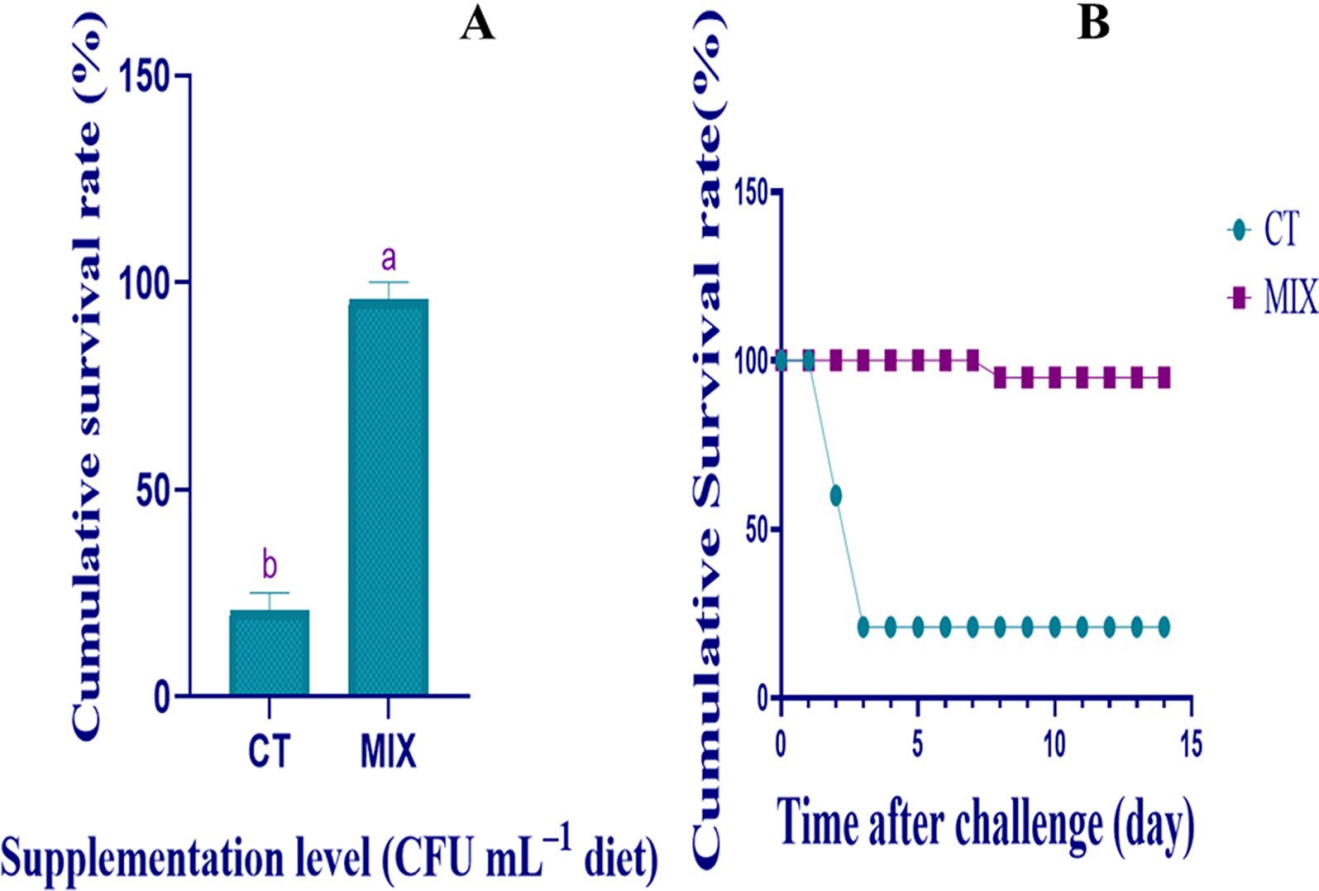
(**A**,**B**) Influences of dietary supplemented autochthonous strains mixture on the cumulative survival rate (%) after inoculation with *Vibrio* *harveyi*. Results are articulated as mean ± standard error (M ± SE). Based on independent T-tests (*P* < 0.05), letters a and b indicate significant differences. *MIX* autochthonous strains mixture concentration at 1 × 10^12^, *CT* Control. n = 24. As shown in (**B**), the cumulative survival rate (%) of cobia after inoculation with *Vibrio* *harveyi* is displayed day-by-day.

The challenge test exhibited that the 70 days application of autochthonous strains mixture improved the protection against microbial infection (Fig. 4A,B). The cumulative survival rate was computed within two weeks. The results showed that feeding autochthonous strains added diets induced significantly lower death against the pathogens, *V.* *harveyi*. A noticeable significant difference (*P* < 0.05) in the cumulative death rate between the control and trial groups fed was observed. The fish mortality in a group fed deprived of additives transpired between 24 and 72 h subsequently injection of pathogens, *V.* *harveyi* at 1 × 10^8^ CFU/mL whilst the only mortality occurred in the trial group after injection of pathogens, *V.* *harveyi* was witnessed 192 h (7 days). The survival percentage was 95.83% for MIX and 20.83% for CT at the end of the challenge test.

### Changes in microbial community diversity

**Figure 5:**
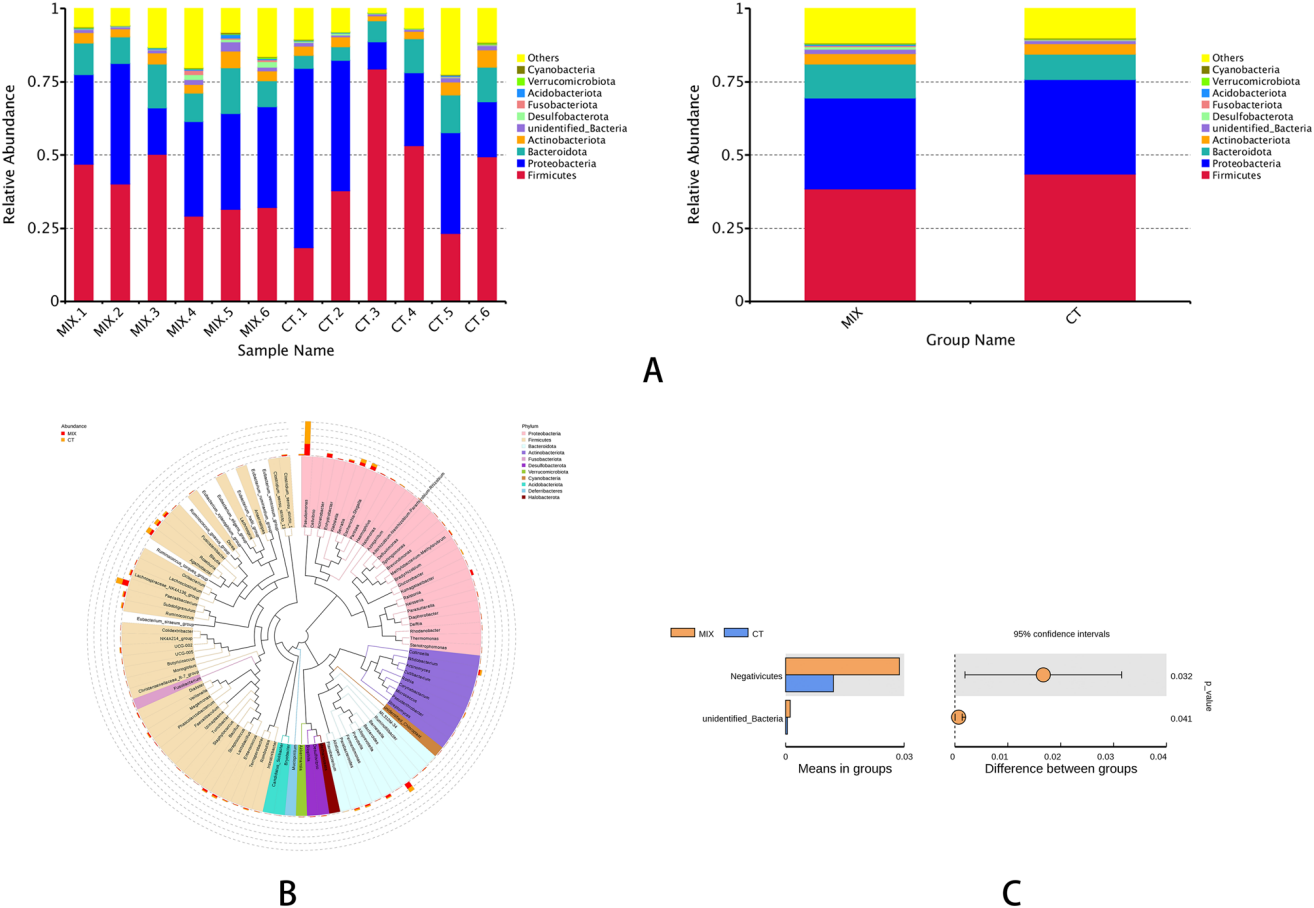
(**A**,**B**)Taxonomy of phyla and genus in intestinal samples. *CT* control group, *MIX* indigenous isolate mixture supplementation group. (**C**) T_test analysis of species differences between groups. Description: the figure on the left shows the abundance of different species between groups. Each bar represents the mean of each group of species with significant differences in abundance between groups. The figure on the right shows the difference confidence between groups, with the left end of each circle representing the lower 95% confidence interval of the mean difference and the far right end of the circle representing the upper end of the 95% confidence interval of the mean difference. The centre of the circle represents the mean difference. The circle colour represents a group with a high mean. The far-right end of the presented results is the intergroup significance test p-value for the different species.

*Firmicutes*, *Proteobacteria*, *Bacteroidetes*, and *Actinobacteria* were the predominant bacteria phyla composing the gut microbiota of juvenile cobia fed supplemented diets or without supplemental diets, which comprised *Firmicutes* (38.35–43.56%), *Proteobacteria* (31.16–32.27%), *Bacteroidetes* (8.75–11.48%), and *Actinobacteria* (3.53–3.71%), respectively (Fig. 5A). In supplemented fish, the highest relative abundant phylum (38.35%) was *Firmicutes*, followed by *Proteobacteria* (31.16%), *Bacteroidetes* (11.48%), and *Actinobacteria* (3.71%). In fish without supplementation, dominant phyla were *Firmicutes* (43.56%), *Proteobacteria* (32.27%), *Bacteroidetes* (8.75%), and *Actinobacteria* (3.53%). Moreover, in fish receiving supplementation, the presence of pathogenic bacteria such as *Pseudomonas* and *Agathobacter* was reduced. In contrast, abundance of known probiotic genera such as *Akkermansia,* *Alistipes,* *Bacteroides,* *Bifidobacterium,* *Blautia,* *Eubacterium,* *Faecelibacterium,* *Lactobacillus,* *Roseburia,* *Ruminococcus* was increased in fish fed the supplemented diet (Fig. 5B). Significant differences (*P* < 0.05) in gut microbiota diversity were observed between fish fed the supplemented diet and non-supplemented. The average abundance of negativicutes in trial (MIX) was 2.9%, and the average abundance in control group (CT) was 1.2%, and the significant p-value of this species between the two groups was found to be 0.0319. (Fig. 5C). For each group, the Alpha diversity index was calculated to assess the effects of the isolates on the abundance and diversity of juvenile’s cobia. The Chao1 index was used to analyze the abundance of the flora. Shannon and Simpson indices were used to examine the diversity of the flora (Table 5). Compared to the control group, there was no significant difference (*P* > 0.05) in the Chao1 and ACE indexes but there were differences in numbers. The Shannon and Simpson index has shown significant differences between the trial and control group.

**Table 5:** Alpha diversity index of gut microbiota of juvenile cobia.

Groups	OTU number	Alpha diversity index			ACE	Goods coverage
		Chao1 index	Shannon index	Simpson index		
MIX	2140.67 ± 168.08	2747.61 ± 191.48	7.65 ± 0.29*	0.97 ± 0.01*	2895.06 ± 189.22	0.97 ± 0.00
CT	1730.67 ± 224.56	2299.71 ± 267.71	6.30 ± 0.38	0.91 ± 0.03	2566.42 ± 288.28	0.97 ± 0.00

*Indicates a significant difference (*P* < 0.05). Data are expressed as mean ± standard error (M ± SE). Numbers in the same row with no superscripts do not differ significantly (*P* > 0.05) by Independent_sample T test.

### Changes in metabolic profiles

**Figure 6:**
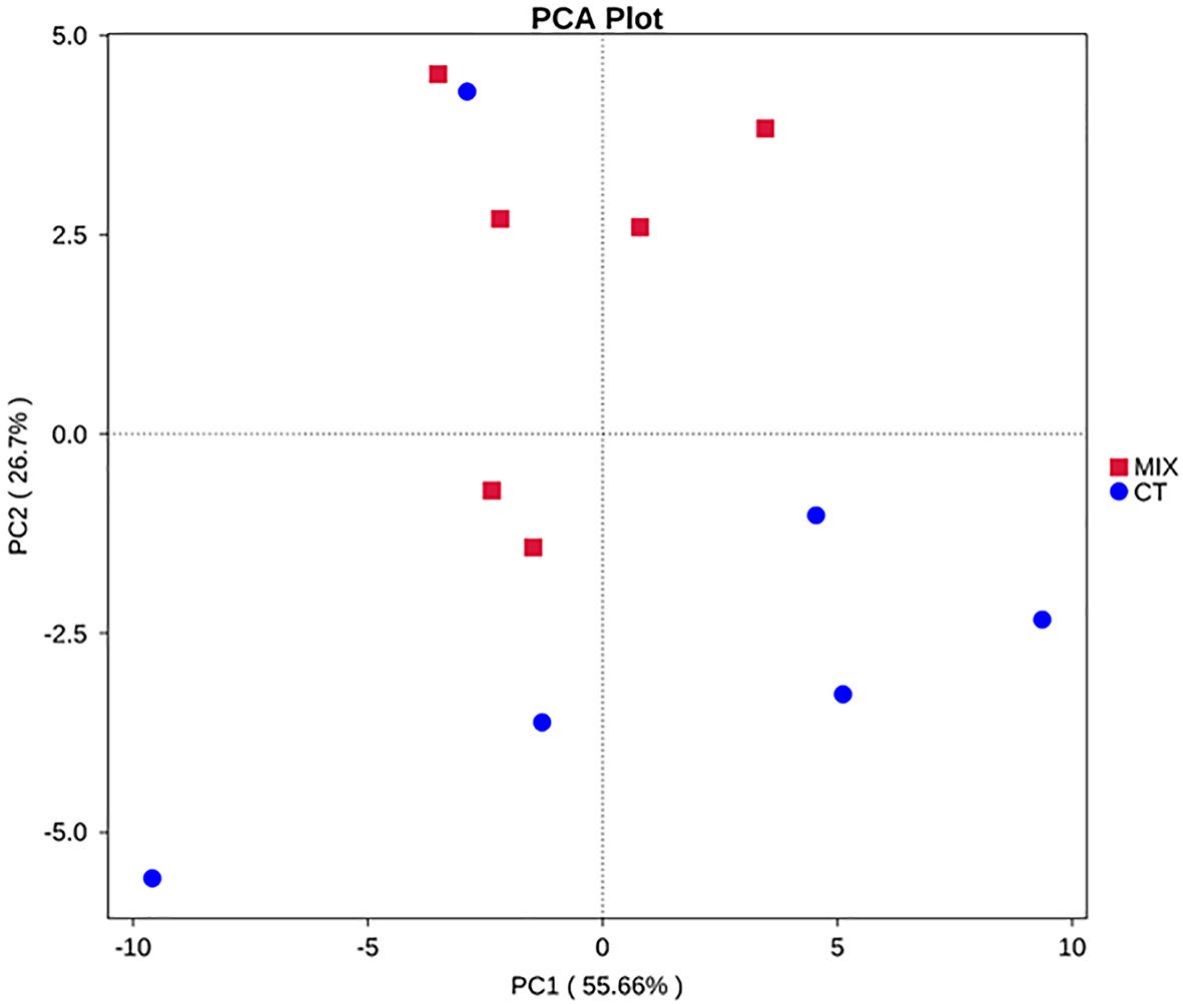
Indigenous isolate mixture fed juvenile cobia was separated from the group without supplementation in metabolite profiles by PCA.

**Figure 7:**
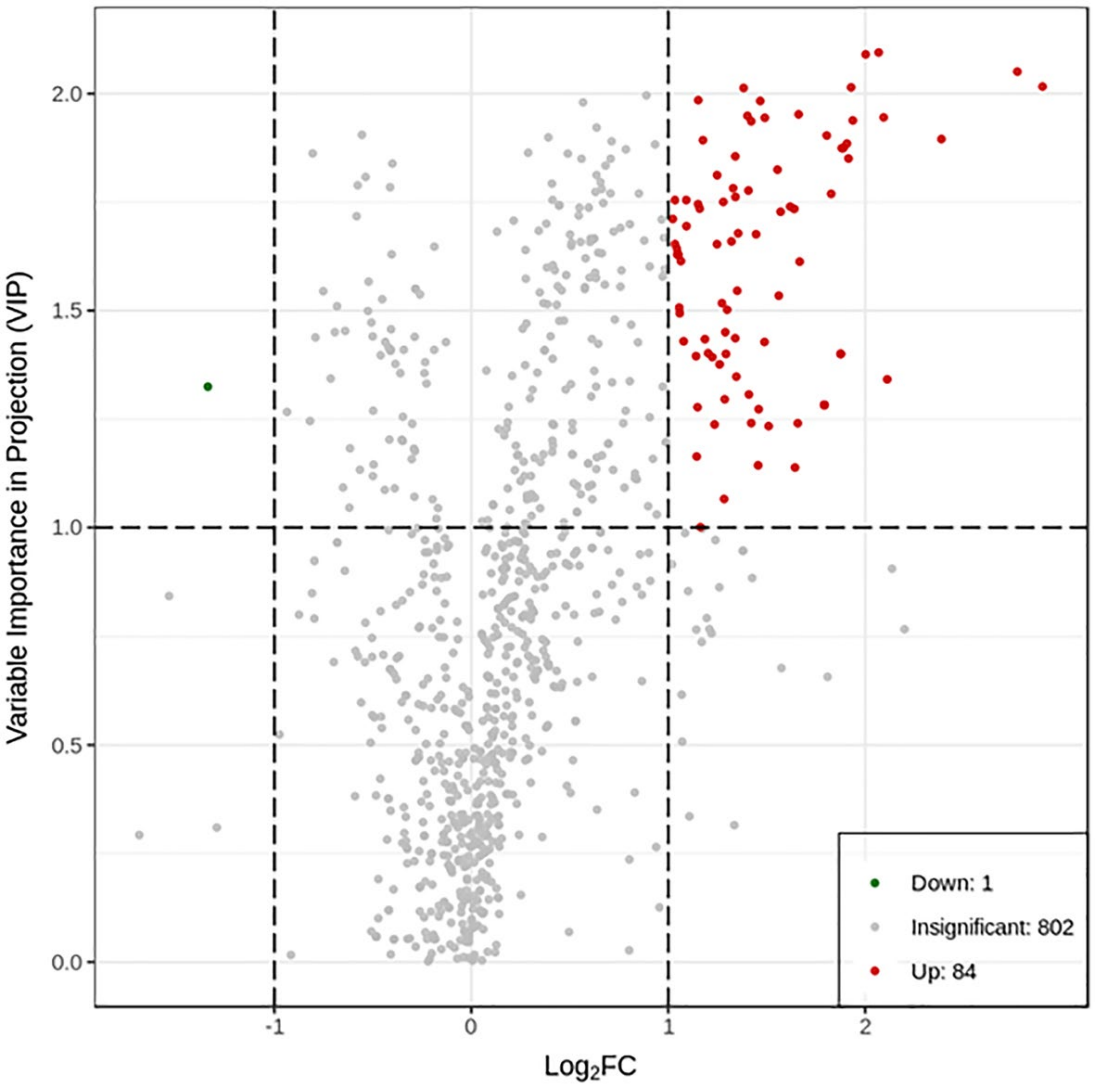
Indigenous isolate mixture treatment changed the metabolic profile of faecal samples of juvenile cobia, with 85 molecular structures being significantly altered compared with controls (fold change ≥ 2 and fold change ≤ 0.5).

Fingerprints of gut metabolites in fish fed the supplemented diet and non-supplemented were different (Fig. 6); the first and the second PCA components (PC1 and PC2) explained differences between the metabolic profiles of fish fed the supplemented diet and the control group. The model constructed in this study for R2X was 0.418, R2Y was 0.913, and Q2 was 0.692, hence the model could be described as effective (Supplementary Fig. 2). Overall, 85 significantly differential metabolites were identified**,** among which 84 were upregulated and 1 downregulated (Fig. 7 and Supplementary Table 1). In addition, P-value or fold change of univariate analysis were combined to characterise differential metabolites further considering FC ≥ 2, VIP > 1, and *P* value < 0.05; 61 metabolites in fish fed the supplemented diet were significantly altered compared with the control group (Supplementary Fig. 3). Differential metabolites with higher FC are shown in Table 6; among upregulated metabolites were included *N*-acetyl-l-glutamine (NAQ), lactose, lactulose, pyridoxal-5-phosphate (P5P), l-tryptophan, d-(+)-cellobiose, *N*-acetyl-5-hydroxytryptamine; the downregulated metabolite was 5′-adenylyl sulfate (APS).

**Table 6:** Polymerase chain reaction primer sequences for real-time reverse transcription (RT-qPCR).

Target genes	Primers (5′ → 3′)	Primer length (bp)
F-Igm-F	CAAGCCATCCGTTGACAAAGT	133
F-Igm-R	TATCCCCTTGCTCCATTCGT	
F-Tcrβ-F	TGGAGCAGGTGTGAAGGTAG	138
F-Tcrβ-R	CCACCGAAGTAAGCCTCTCG	
F-Csfr1-F	GGAGTTCTGAGCTGGTGGTC	135
F-Csfr1-R	AGGTGCGGACATTGCCATTG	
F-MHC-I-F	GCGTTGGCAGTGACACATTC	151
F-MHC-I-R	AGTCCTGTTTACCCTCTGCT	
F-MHC-2-F	ATTGCAGTCACGTACCTGTC	132
F-MHC-2-R	CGTCAATCACCAACCTGTGC	
β-actin: F	AGGGAAATTGTGCGTGAC	114
β-actin: R	AGGCAGCTCGTAGCTCTT	

Where β-actin is cobia β-actin, *IgM* indicates Immunoglobulin, *Csfr1* Colony-stimulating factor receptor 1, *MHC-1* Major histocompatibility complex 1, *MHC-2* Major histocompatibility complex 2, *eflα* elongation factor 1-alpha, *Tcrβ* T cell receptor beta.

**Figure 8:**
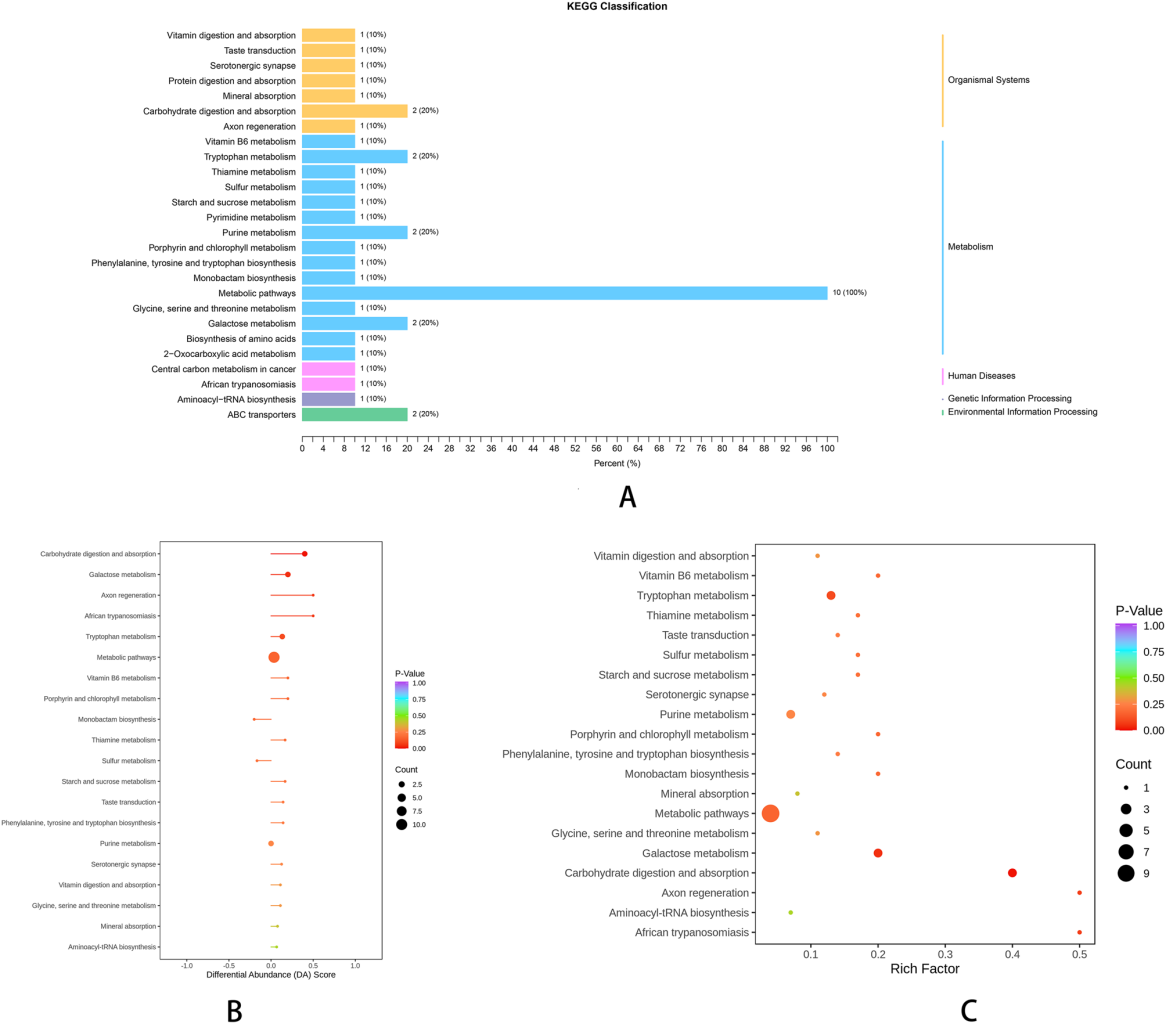
(**A**) Pathway classification map of differential metabolites. The ordinate coordinate is the name of the kegg metabolic pathway, and the abscissa is the ratio of the differential metabolites annotated to the pathway and the number of their number to the total number of metabolites on the annotation. www.kegg.jp/kegg/kegg1.html. For previous uses, the Kanehisa laboratory have happily provided permission. (**B**) Variance abundance score chart. The ordinate coordinate represents the difference pathway name, and the abscissa represents the difference abundance score (DA Score). The DA Score reflects the overall change in all metabolites in the metabolic pathway, with a score of 1 indicating an upward adjustment in the expression trend of all identified metabolites in the pathway, and a score of − 1 indicating a downward revision of the expression trend of all identified metabolites in the pathway. The length of the segment represents the absolute value of DA Score, the size of the dot at the end of the segment indicates the number of differential metabolites in the pathway, the dots are distributed on the left side of the central axis and the longer the segment, the more inclined the overall expression of the pathway is to be downregulated, the dots are distributed on the right side of the central axis and the longer the segments, indicating that the overall expression of the pathway is more inclined to upward adjustment, and the larger the dots indicate the greater the number of metabolites. The line segment and dot color reflect the size of the P value, the closer to red means that the P value is smaller, and the closer to purple means that the P value is larger. (**C**) Differential Metabolite Kegg Enrichment Map. The abscissa coordinate represents the Rich factor corresponding to each channel, the ordinate coordinate is the channel name, the color of the dot is P-Value, and the redder the enrichment, the more significant the enrichment. The size of the dots represents the number of differential metabolites enriched. www.kegg.jp/kegg/kegg1.html. For previous uses, the Kanehisa laboratory have happily provided permission.

**Figure 9:**
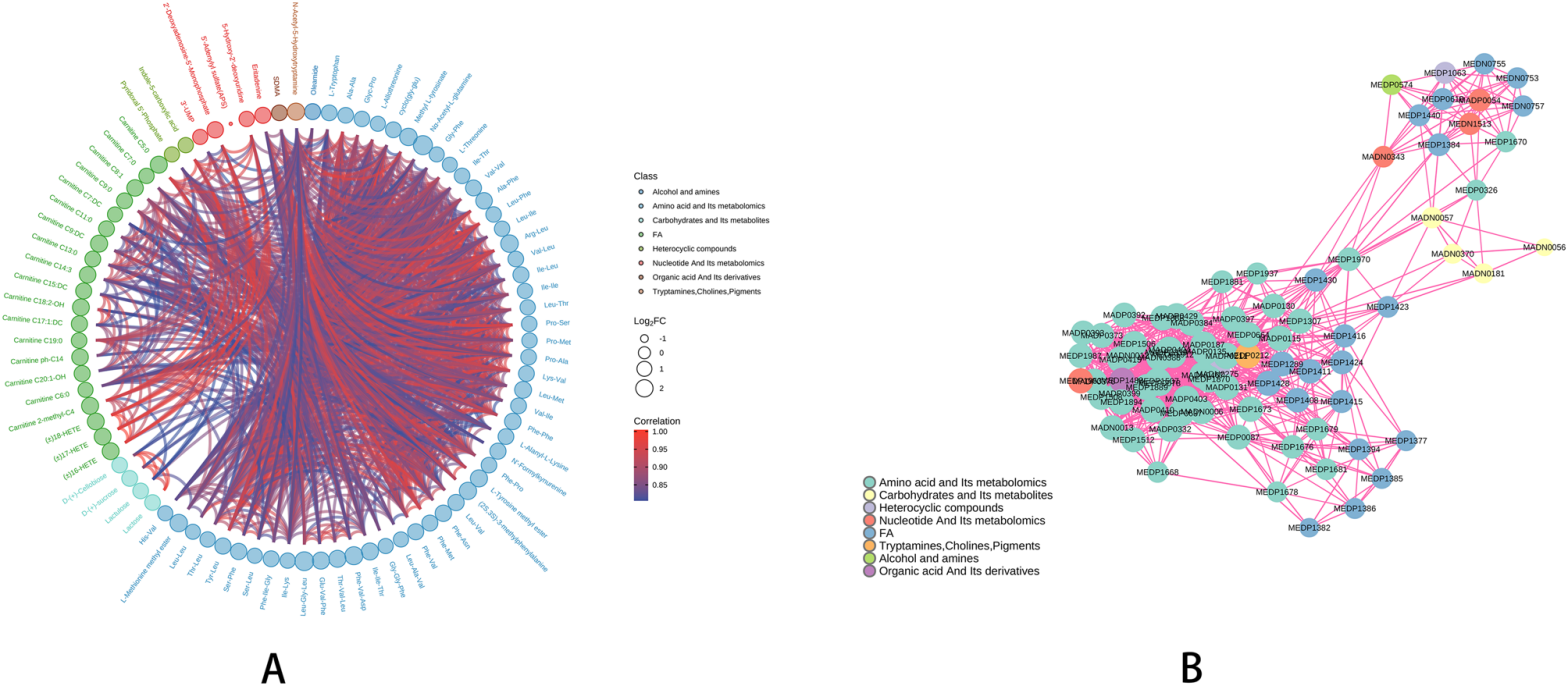
(**A**,**B**) Correlation analysis of differential metabolites. (**A**) Differential metabolite chord plot. The outermost layer in the figure is the name of the differential metabolite, and the size of the dot represents the log of the corresponding differential metabolite. The size of the FC value; the different colors represent the different classes of the corresponding differential metabolites; the wiring represents the size of the Pearson correlation coefficient between the corresponding differential metabolites, the pink lines represent the positive correlation, and the blue lines represent the negative correlation. The default pair is |r| Differential metabolite pairs > 0.8 and *P* < 0.05 are plotted. (**B**) Differential metabolite correlation network diagram. The points in the figure represent significant difference metabolites, the size of the points is related to the degree of connection degree, the larger the point, the greater the degree of connection, that is, the more points (neighbors) are connected to it. Pink lines represent positive correlations and blue lines represent negative correlations. The thickness of the line represents the size of the absolute value of the correlation coefficient, and the thicker the line, the greater the correlation. The default is |r| Differential metabolites > 0.8 and *P* < 0.05 are plotted.

Cluster analysis of differential metabolites was conducted to assign metabolites to their corresponding biological processes. Hierarchical cluster heatmaps are displayed in Supplementary Fig. 3A,B for individual molecules or features identified within each cluster. Based on KEGG annotation results (Fig. 8A), differential metabolites were assigned to the following categories: metabolism, galactose metabolism, tryptophan metabolism, carbohydrate digestion and absorption, purine metabolism, and ABC transporters. A separation of metabolic profiles was found between supplemented fish and the control group without supplementation. Alterations in gut microbial community pathways were observed pathways associated with carbohydrate and its metabolites, nucleotide and its metabolomics, amino acid and its metabolomics, heterocyclic compounds, and tryptamines, cholines, and pigments (Fig. 8B,C). Moreover, metabolic proximity was observed between significantly different metabolites associated with alcohol and amines, carbohydrate and its metabolites, nucleotide and its metabolomics, amino acid and its metabolomics, fatty acid (FA), heterocyclic compounds, and tryptamines, cholines, pigments (Fig. 9A,B).

### Association between gut microbiota composition and metabolic profiles

Pearson correlation coefficient (PCC) was used to evaluate functional relationships between variations in gut microbiota composition and metabolic profiles (Supplementary Fig. 4). PCC results revealed that changes in gut microbiota were associated with altered metabolic profiles. Overall, 200 metabolites displayed an association with particular gut microbiota, revealing the functional relationship between gut microbiota and metabolic profiles. The hierarchical clustering of Spearman correlation coefficients of differential microorganisms and metabolites showed a positive correlation (Supplementary Fig. 5). Similarity percentage analysis (Simper) is based on the decomposition of the Bray–Curtis difference index, which enables quantification of the contribution of each species to differences between the two sample groups. Simper analysis was conducted using the simper function in the vegan R package. As shown in Supplementary Fig. 5, *Firmicutes* and *Proteobacteria* were widespread, indicating that these phyla were key to determining separation of metabolite profiles. However, certain metabolites could not be assigned to any KEGG pathways based on their structure and their association with gut microbiota composition. Collectively, the findings of the present study revealed a significant interdependence between gut microbiota composition and metabolic profiles in juvenile cobia.

## Discussion

The present study indicated that the diet supplemented with a mixture of autochthonous strains improved the specific growth rate and weight gain and reduced the feed conversion ratio in juvenile cobia compared with the group without supplementation. This boost in growth performance could be accredited to the improved digestibility of nutrients. The present study's blood serum parameters studied and documented are generally within the applicable limits. These blood serum biochemical indices are recognized as parameters that show the overall fish health status. The significant proteins are globulin, Serum proteins, and albumin^30^. There were no significant differences in AST, ALT, TG, CHO, and GLU in our present study,nevertheless, albumin, total protein, and globulin content were significantly higher compared to the control group. The result implied that supplemented diet utilizing a mixture of autochthonous strains enhanced juvenile cobia immunity. Similar results were reported in rainbow trout (*Onchorchyncus* *mykiss*)^31^, juvenile Jian carp^32^ when feed additives probiotics were utilized. Tahmasebi-Kohyani et al.^33^ reported that in the innate immune system, the additives could impact cellular and humoral components may enhance lysozyme activity and serum complement of fish.

In fisheries and aquaculture, the insertion of the haematology procedures has become a treasured knowledge in accessing the health of fishes by fishery biologists^34^. The utilization of probiotics to enrich immunological activities and influence the haematological indices in fish species has been explored^35^. Actual actions of blood cells assist their passage via blood paths to the infection sites^36^. The white blood cells (WBC) are notable as unique significant defence factors of the body, increasing speedily upon infections. The elevation in white blood cells, red blood cells, corpuscular haemoglobin concentration, mean corpuscular volume, haemoglobin, and mean corpuscular haemoglobin owing to isolated bacteria supplementation shows that the mixture of autochthonous strains has anti-infection properties or pathogens^37^. Due to isolated bacteria feed, the red blood cells tally improved, pointing to an immunostimulant influence^38^. The present results are similar to those reported by Refs.^39,40^. Besides, the contents of haemoglobin in the blood samples were higher compared to the group without supplements, an indication that the conveyance of oxygen-enhanced the well-being of the fish was improved and subsequently improved the growth and immunity of the fish. In this study, serum glucose concentration considerably decreased in fish fed with supplemented diets. The serum insulin level is generally improved via reducing blood glucose with diet additives^41^. The beneficial influence of supplemented isolates is that they were able to fuel the insulin activity, therefore decreasing the glucose level in the blood.

The serum total protein, albumin, and globulin in juvenile cobia significant changes reflect in the supplemented groups in the present study. The globulin contents are unquestionably crucial for sustaining the immune functions in the blood and a hale and hearty immune system. Nonetheless, albumin is essential for supporting osmotic pressure necessary for properly circling body fluids and saving as a carrier of plasma^42^. The rises or enhancement of the contents of globulin, albumin, and the total serum protein are thought to reveal robust and resilient innate immunity^42,43^. The current study shows no changes in cholesterol and triglycerides, which could improve the fish's cardiovascular action, reflecting positive influence in terms of the welfare of fish. A more in-depth investigation will be necessary to identify the precise mechanism responsible for the non-significant increases or decreases observed in this study. There is an acceptable line between effectively achieving benefits and achieving undesirable outcomes when choosing the type and number of probiotic bacteria for juvenile cobia. With immunostimulants usage, fish's serum complement and lysozyme activity could be enhanced^43,44^. Our results indicated an increase in activity of lysozyme in blood serum of juvenile cobia with a supplemented mixture of autochthonous strains. The present study submits that utilizing a mixture of autochthonous strains might conceivably safeguard fish from possibly invasive microorganisms.

The body encompasses a multifaceted antioxidant protection web that depends on non-enzymatic antioxidants and endogenous enzymatic. These molecules mutually act against free radicals to repel detrimental effects on essential biomolecules and, eventually, body tissues^45,46^. Depending on the response of these molecules to an invasion of ordinary free radicals, they could be characterized as first, second, third, and even fourth-line defence antioxidants. The efficacy and role of glutathione peroxidase (GPX), superoxide dismutase (SOD), and catalase (CAT) which essentially belong to the first line defence antioxidants, is vital and crucial for the whole antioxidants defence approach, particularly in reference to superoxide anion radical (O_2_^−**·**^) that is eternally produced in normal metabolism of the body, mainly via the "mitochondrial energy production pathway (MEPP)"^45,46^. A number of studies have been carried out in relation to antioxidants and their importance of the attendant cellular damage and averting oxidative stress, especially the essential role of GPX, CAT, and SOD. After ten weeks of supplemented feed of autochthonous strains, the current study analyzed CAT, GPX, MDA, and SOD enzyme activities in the liver and head kidney in juvenile cobia. Superoxide dismutase and catalase are the key antioxidant enzymes tangled in hunting extreme ROS and guarding species from oxidative destruction^47^. Higher SOD, GPX, and CAT activities were detected in supplemented groups in our research. Similar results were reported by Abarike et al.^48^. The boosted antioxidant activities suggested higher antioxidant ability to reduce oxidative stress succeeding production of ROS via immune cells, and enhancement of cobia physiological performances.

The assessment of the existence of the digestive enzyme and their activities levels are utilized as a comparative food acceptance indicator, the frequency of fish development, digestive aptitude, and survival rate of fish^49^. In accordance with Solovyev et al.^50^, the trophic niche of fish in their natural conditions and feeding ecology is indicated by digestive enzymes activities. In this study, the results suggested an improvement of digestive enzymes activities such as amylase, lipase, cellulase, pepsin, and trypsin activities enhanced growth rate in fish could be attributed to the fish's ability to adequately processed food that allowed high assimilation rates and satisfactory exceedingly active digestive enzymes^51^. The digestion of cellulose, carbohydrates, lipids, and protein could be attributed to the involvement of lipolytic, cellulolytic, amylolytic, and proteolytic enzymes^52,53^, therefore, are accountable for stimulating the cultured fishes development and growth^54^. Also, the digestion rate affects the uptake of nutrient, and ultimately the growth of the fish are affected^55^.

Generally, the improved immune response as a result of the mixture of autochthonous strains additive is necessary to promote the immunity of fish. Besides, dietary supplementation in this study with a mixture of autochthonous strains additive improved juvenile cobia disease resistance, as shown in the challenge study. The mixture of autochthonous strains additive-fed group displayed the highest survival percentage post-challenge. *Vibrio* *harveyi* is a common pathogenic fish disease in the aquaculture industry. Many studies have proven that fish fed with dietary additives displayed more excellent resistance to pathogenic challenges^48,56–61^.

Several authors have assessed the immunostimulant properties of diverse compounds through immune-related genes expression studies. The assessment of the regulation of the expression of six immune-related genes (*igm,* *tcrβ,* *csfr1,* ef1α, *mhc-1*, and *mhc-2*) in the midguts of cobia was carried out in the present study using quantitative RT-PCR. Our results exhibited a significant increase in *igm,* *csfr1,* and *mhc-1* gene expression within the investigational period. Immunoglobulin (IgM) is recognized as the first antibody to appear in reaction to introduction to an antigen in fish. After ten weeks, our study has exhibited a significant up-regulation of *igm* gene expression. Similar results were reported by Reyes-Becerril et al.^62^ in toleopard grouper (*Mycteroperca* *rosacea*) and Bahi et al.^26^ in gilthead seabream (*Sparus* *aurata* L.).

As has been well recognized, the critical humoral adaptive immune reaction elements are the Igs. Immunoglobulin (IgM) epitomizes the key Ig in the teleosts plasma and the core player in systemic immune reactions. Immunoglobulin is also tangled in reactions against numerous pathogens^63,64^. The *igm* genes encrypt the antibodies, a defence proteins family, which seems distinctive in almost all vertebrates. The antibodies are produced via B-cells. The *igm* gene is expressed in a tissue-specific fashion. It goes through a multifaceted series of chromosomal reorganizations to "generate a large repertoire of functional antigen-binding sites and somatic mutations to permit structural refinements of the antigen binding site," hence producing high-affinity antibodies^65^. The upregulation of the *igm* in the present study suggests that the isolates supplementation help improve the immune system of the juvenile cobia, and this could be the reason for the higher survival rates witnessed in the challenge results.

*mhc* genes have enticed huge attention as significant features of adaptive immunity in vertebrates. *mhc* *2* genes are vital members of the MHC family and play a key role in binding foreign peptides resulting from extracellular pathogens and releasing them to support T cells^66^. Even though there are many studies of *mhc* genes in teleost^25,67^, materials are still inadequate that teleost *mhc* genes play a part in immune defence thru infection with diverse pathogens. In the present study, the expression patterns of *mhc* *1* and *mhc* *2* genes in the midguts of cobia have been investigated after 10 weeks of autochthonous strains supplementation. The real-time PCR investigation revealed that cobia *mhc* *1* mRNA was up-regulated in the supplemented group, and *mhc2* has shown no significant difference compared to the control group. The up-regulation in the intestines indicates that the fish immune system could respond when pathogenic antigens move in Refs.^25,67^. Earlier, researchers have reported that *mhc* genes are accountable for stimulating specific immune reactions in the direction of the foreign pathogens^68,69^. The present results of *mhc* in cobia could be a demonstration of the vital immune function of up-regulated *mhc* *1* gene.

The gut microbiota is considered a vast and complex ecosystem in the body. Changes in the gut microbiota are closely associated with many factors including feed, genetic predisposition, lifestyle, and environment and individual hosts. Moreover, gut microbiota plays a vital role in maintaining intestinal health, promoting intestinal development, resistance to bacterial infections, regulation of energy absorption, and lipid metabolism^70^. Bacterial communities in the digestive tract are vital components of the host mucosal barrier defences^13^. Competition for adhesion sites and nutrients may limit or decrease the abundance of pathogens in the gut. Various compounds may also be produced to counteract the presence of pathogens, including hydrogen peroxide (H_2_O_2_), organic acids, bacteriocins, siderophores, and antimicrobial peptides. Notably, the gut microbiota community is also composed of species that can act as opportunistic pathogens, infecting immunocompromised hosts and/or causing secondary infections. Therefore, such communities can impact host development. By the alpha diversity analysis, dietary supplementation of a mixture of autochthonous bacterial strains significantly increased the diversity of bacterial species. However, there was no difference between the dietary supplementation group and the control group with respect to species richness. There may be a reason for this depending on the type of fish, the fish's eating habits, and the fish's age and size. In the intestines of marine carnivorous fish, Bacteroidetes, Proteobacteria, Fusobacteria, Firmicutes, and Actinobacteria are dominant phyla^71^. Researchers have reported Proteobacteria, Firmicutes, and Bacteroidetes to be the three most dominant phyla in the gut flora, with relative abundances exceeding 70%^72,73^. According to Costantini et al.^74^, Firmicutes enhance piscine immunity, digestibility, and resistance to pathogenic bacteria. Bacteria in the proteobacteria family are the most dangerous, including *Vibrio* *cholera*, *Escherichia* *coli*, and *Rhodocyclus*. This increase in prevalence could be a sign of dysbiosis, metabolic and nutritional disorders, and disease risk^75^. Bacteriodetes can enhance the host's immune system, improve the gut's mucosal barrier, and decompose polysaccharides^76^. Based on the results of this study, Firmicutes were the most abundant phylum among the three and Proteobacteria and Bacteroidetes were significantly augmented following the consumption of autochthonous strains. Supplementation with autochthonous strains changed gut microbiota composition, altering microbial abundance in the gut by reducing the load of pathogenic bacteria and improving that of probiotic species exerting positive impacts on the intestinal microbial composition of the juvenile cobia.

Several studies have reported that probiotic supplementation leads to changes in the composition of gut microbiota in cultured aquaculture species, thus altering gut metabolism. Microbes in the digestive tract have been shown to affect appetite/ingestion, digestion, and metabolism while specific metabolites, for example, short-chain fatty acids (SCFAs), indoles, propionate, butyrate, and acetate and influence digestion and metabolism^77^. Enterocytes are stimulated by these metabolites, which can lead to changes in their intestinal barrier function, a change in absorption capacity, along with nutrient uptake and storage in the gut, for example, changes in enzyme activity and fat storage, affects metabolism^77,78^. In a few fish species, the gut microbiome has been examined for its effect on metabolism. As the composition of microbiota changes in the present study, a number of metabolisms and metabolic pathways change. In our study, *N*-acetyl-l-glutamine (NAQ), lactose, and lactulose, pyridoxal-5-phosphate (P5P), l-tryptophan, d-(+)-cellobiose, small peptides, and other metabolites, were significantly upregulated in the gut of juvenile cobia after diet supplementation with autochthonous bacterial strains.

*N*-acetyl-l-glutamine appears to possess a metabolic fate comparable or similar to free glutamine^79^. Collectively, these results show that *N*-acetyl-l-glutamine plays a crucial role in maintaining the function of animal immune system. In the present study, *N*-acetyl-l-glutamine was found to be significantly upregulated in the gut of juvenile cobia after diet supplementation with autochthonous bacterial strains, suggesting that it may play a vital role in juvenile cobia. Lactulose is an artificial, non-digestible sugar used to treat chronic constipation and hepatic encephalopathy, and is formed by the isomerisation of lactose. Lactose is formed from fructose and galactose, whereas lactose is primarily found in milk and composed of glucose and galactose. Nutritional lactose plays a critical role in enhancing juvenile animals' growth performance, intestinal health, and overall well-being. Part of nutritional lactose is fermented in the gut by microorganisms such as *Lactobacillus* spp., thus generating lactic acid and negligible amounts of acetate, which contribute to maintaining intestinal acidity in animals^80^. Lactose and lactulose are prebiotics and have health benefits by selectively stimulating lactobacilli and bifidobacteria in the gut of mammals with reduced lactose assimilation and free from intestinal disorders. Other studies suggested that the health benefits of lactose as a prebiotic are related to the gut microbiota composition and with several bacterial metabolites including butyrate, which is an essential source of energy for the intestinal mucosa as well as acts as an anti-inflammatory, amongst other health benefits^81^. Energy is required to support continuous growth in fish, since maintenance only accounts for 10% or less of carbohydrate requirement. Lactose serves as the main carbohydrate in young organisms. Insufficient lactose and lactulose ingestion in fish could lead to immune function deficiency and delayed or hindered growth. In the present study, diet supplementation with autochthonous bacterial strains enhanced distribution of lactose and lactulose metabolites in the gut of juvenile cobia. Therefore, growth performance verified in a previous study could result from adequate lactose and lactulose ingestion provided by the presence of added autochthonous strains.

Cellobiose is formed when cellulose is partly hydrolysed by the enzyme cellulase. Certain microbes (such as *Bacteroides* *cellulosolvens)* possess cellulases and other enzymes able to metabolise cellulose into cellobiose^82^. However, the physiological functions of cellobiose are still poorly understood. In the present study, d-(+)-cellobiose was upregulated in juvenile cobia following supplementation with autochthonous bacterial strains. However, the exact roles that can be attributed to cellobiose in juvenile cobia could not be elucidated in the present study. To understand how cellobiose controls or modulates gut inflammation, concentration of SCFAs in faeces should be measured and associated with modifications in gut microbiota of juvenile cobia. It is known that the liver regulates P5P metabolism, but more recent evidence indicated that intestinal cells play a considerable role in vitamin B6 metabolism^83^. Therefore, gut ecosystem and liver health are crucial for sufficient P5P availability^84^. The ingestion and supplementation of vitamin B6 promoted certain immune functions in vitamin B6-deficient animals and humans. Hence, the significant upregulation of P5P in the present study is beneficial to the immune system of juvenile cobia.

Besides its dietary importance, nutritional protein has other health benefits owing to the release of bioactive peptides. Bioactive peptides are inactive but specific amino acids within the parent protein and must be released to exert their potential biological activities. Based on amino acid composition, sequence, charge, and length, bioactive peptides could exhibit promising biological properties, including immune and endocrine booster, anti-inflammatory, antimicrobial, antioxidant, and immunomodulatory^85^. Particular protein fragments, which amend or boost the host’s defence response, comprise adaptive and innate reactions, and eventually can protect the organism against harmful microorganisms^86^. However, the molecular mechanism underlying the immunomodulatory function of bioactive peptides is still not fully understood. Nonetheless, bioactive peptides can lead to substantial physiological changes at the tissue level, including improving eradication or proliferation of T and B cells, triggering activity of natural killer cells and macrophages, and controlling the release of immunoglobulins^86,87^. Certain amino acids often occur in peptides with immunomodulatory properties, including hydrophobic amino acids (phenylalanine, valine, proline, leucine, and glycine) and aromatic and negatively charged amino acids^86^. In addition, bioactive peptides are less likely to accumulate in body tissues without severe problems, hence serving as an alternative to conventional drugs in disease treatment. Bioactive peptides can be used as a pharmaceutical or as an additive in diet with considerable potential effect against infection- and disease-causing agents. In the present study, diet supplementation with autochthonous bacterial strains benefited juvenile cobia by significantly upregulating the release of several small peptides, however, further studies are required to elucidate the exact mechanisms underlying the role of the small peptides found to be upregulated in the current study in juvenile cobia immune system.

## Conclusion

Healthy growth is dependent on maintaining an appropriate microbial ecology on the host. Probiotics, which are large species of bacteria that supplement the host, are an alternative to antibiotics. There has been tremendous progress in the field of probiotics, even though their mechanisms of action are not yet completely understood. The intestinal barrier is strengthened and the immune system is modulated by them. Despite findings in several biological systems, limited studies have documented autochthonous strains of probiotic effects in fish. No clear standard has been established for how much evidence is needed to support a food's bioactivity. This is a multifaceted issue involving both scientific and regulatory considerations. It is not surprising, then, that the commercial use of probiotics has continued because consuming probiotics in well-defined amounts in foods poses very little risk and can have a host of benefits.

Our results highlight the immuno-prospective and therapeutic of a mixture of autochthonous strains *Bacillus* sp. RCS1, *P.* *agglomerans* RCS2, and *B.* cereus RCS3 to safeguard fish from infectious diseases caused via pathogenic microorganisms and save fish survival and growth enhancers. As evident in the present study, the inhibition of microbial infections, non-specific immunity, and the immunity of fish in aquaculture systems may be accomplished by utilizing a mixture of autochthonous strains as the diet supplement of fish. The improved antioxidant enzyme activities (SOD and CAT), digestive enzyme activities (trypsin, lipase, amylase, pepsin, and cellulose) could be connected with the enhanced resistance of fish against infection of *V.* *harveyi*. Supplementation with autochthonous strains changed gut microbiota composition, altering microbial abundance in the gut by reducing the load of pathogenic bacteria and improving that of probiotic species. Furthermore, diet supplementation with autochthonous strains modified the intestinal metabolic profile thus boosting immunity and growth of juvenile cobia. However, functional studies are required to elucidate the mechanisms underlying the roles of compounds found to be up-regulated in the current study. The results provide a further understanding of fish microbiome concerning composition, metabolite content, and the potential effect on the host gut condition in response to probiotics supplementation. The present study also contributes vital information to the general understanding of structure and composition of microbiota in vertebrate organisms.

## Materials and methods

### Autochthonous strains preparation

The autochthonous strains were isolated from the digestive tract of juvenile cobia in our earlier study^2^, the isolates were stored in a − 80 °C refrigerator until usage. The autochthonous strains were prepared following the procedures defined by Amenyogbe et al.^88^ with a slight modification. The cell suspensions were activated in luria broth at 37 °C for 48 h. Then, microorganisms were centrifuged at 5000 rpm (Beckman Coulter, AK, USA) for 10 min. After, sterile phosphate-buffered saline (PBS) was used to wash the strains three times. They were then freeze-dried and re-suspend in PBS. After, the viable cells were evaluated through the spread plate technology based on cell contents determined with OD600 that exhibited linear proportion to the viable cell numeral within the following suspension. Lastly, the values of OD600 measured in cell suspension (1.5) were adjusted to the satisfactory value (CFU/mL) before the ensuing diet supplementation trials. The supplemental feed was top-coated with a mixture of autochthonous strains at a 1 × 10^12^ CFU/mL diet based on our piloted work and previous studies^88,89^ the dosage was carefully chosen. Following the methods described by Refs.^10,88–90^, with a slight modification, the viability and concentration of the native isolates mixture in the diet was studied. Concisely, one gram of the supplemented feed was homogenized using 9.0 mL of germ-free PBS and serially adulterated to 10^11^ and 10^12^ respectively. After, spread on Luria broth agar (LBA, Sangon Biotech) media a volume of 0.1 mL in triplicate. The colony counts were assessed weekly from week one to week ten, subsequently incubated for 24–48 h. The previous piloted experimental data on the isolates survivability revealed that the finest viability was within the first week; hence, the diets were supplemented weekly for surety of high levels of autochthonous microorganisms in the supplemented diets. Based on the previous study conducted, the supplementary feed was top-coated with the viable mixture of autochthonous strains at 1 × 10^12^ CFU/mL diet in a 1:1:1 ratio. During this experimentation, the dissolved oxygen concentration > 7.0 mg L^−1^, the salinity 40 ± 1 g L^−1^, the water temperature was at 27 ± 1 °C, and pH 8.0 ± 1. To have precisely one-third of each isolated strain's cells, each strain was diluted serially.

### Fish management and feeding trial

As described by Amenyogbe et al.^88^, a total of 120 mixed-sex juveniles of cobia (*Rachycentron* *canadum*) with (average body weight: 183.867 ± 0.949 g) were acquired from Hainan Blue Ocean Aquaculture Co., Ltd (Hainan province) and then transported to the South Marine Aquaculture Seed Base of State (863) Program, Donghai Island (Guangdong province, China). They (fish) were allowed to be acclimatized for 1 week. Within the acclimatization period, commercial feed (crude protein ≥ 40%, crude fibre ≤ 5.0%, crude ash ≤ 16%, crude lipid ≥ 6%, moisture ≤ 12%, total phosphorus 0.90–1.60 and lysine ≥ 2.10) purchased from (Guangdong Yuequn marine biology research and Development Co. Ltd., Jieyang, China) was used to feed. To group the fish for the trial, they were not fed for 24 h. Randomly, the fish were placed in 6 fibreglass buckets (1000 L, 20 fish/bucket) and divided into two experimental groups [control (*n* = 3, 20 per tank) fed with commercial feed, and MIX groups (*n* = 3, 20/tank] were fed the commercial diet added with a mixture of autochthonous strains with concentration at 1 × 10^12^ CFU/mL, with each group three replicates in a flow-through system. About 80% of the water volume was exchanged daily, and tank water was aerated 24 h/day to ensure a high dissolved oxygen level. For ten weeks, the fish were fed twice a day (8:00 and 17:00) at 10% of their average body weight in continued circulation water.

### Sample collection

On the 70th day of the feeding trial (10 weeks), the fish were not fed for 24 h. The weight gain rate of each tank was calculated based on the total body weight of the fish. In addition, the survival rate was calculated based on the number of fish in each tank. Subsequently, nine fish from each replicate tank were randomly taken, anaesthetized with ethyl-3-aminobenzoate methane-sulfonate (MS-222; Sigma, USA, 100 mg) to abate pain dissected on ice. Every individual fish in each tank taken was weighed and measured for subsequent growth parameters analysis, using the following formulas: Survival rate (SR) = R_t_ × 100/R_0_. Where R_t_ = final number of fish and R_0_ = initial number of fish; Weight gain (WG) = Final weight − Initial weight; Specific growth rate (SGR) = [(LnFinal weight − LnInitial weight)/time] × 100; Feed conversion ratio (FCR) = Food intake/Weight gain^88,89^. Blood was collected and left overnight from the caudal veins of two fish from each replicate tank. Then, their midguts, head, kidney, and liver (vital organs to the body's immune system and metabolic functions) were then collected with a sterile scalpel and packed into 2-ml cryopreservation tubes to analyze digestive and antioxidant enzymes activities and gene expression, respectively. Quickly, all samples were frozen in liquid nitrogen and preserved at − 80 °C until use. The mortalities numbers per replicate were also documented to compute the survival rate. The blood was centrifuged for 10 min at 2000*g* at 4 °C, and the serum was collected for analysis of serum biochemical indices. After another fish was randomly harvested from each replicate tank then dissected on ice. To evaluate haematological parameters, 1 mL of blood from one fish from each replicate was collected using 10% ethylene diamine tetraacetic acid (EDTA). Several parameters were measured by following the methods described by Refs.^88,89^. They included white blood cell count, haemoglobin, red blood cell count, mean corpuscular volume, hematocrit, and mean corpuscular haemoglobin concentration. Randomly, another nine juvenile cobias from each tank were selected and anaesthetised with ethyl-3-aminobenzoate methane-sulfonate (MS-222, Sigma-Aldrich, Saint Louis, MI, USA, 100 mg. Full fish intestines were collected aseptically; six intestinal content (MIX.1 and MIX.2 from Mix1 tank; MIX.3 and MIX.4 from Mix2 tank; MIX.5 and MIX.6 from Mix3 tank; CT.1 and CT.2 from CT1 tank; CT.3 and CT.4 from CT2 tank; CT.5 and CT.6 from CT3 tank was randomly selected and extracted and submitted to gut microbiota and metabolic profiling analysis. All samples were frozen in liquid nitrogen and stored at − 80 °C until further use.

### Serum biochemistry analysis

As described by Amenyogbe et al.^88^, a fully-automated biochemical analyzer (Chemray 240, Shenzhen, China) was utilized with the help of Wuhan servicebio technology CO., LTD (China), to measure the biochemical blood parameters in the present study, comprising alkaline phosphatase (ALP), triglyceride (TG), albumin (ALB), cholesterol (CHOL), total protein (TP), glucose (Glu), Aspartate aminotransferase (AST), and alanine aminotransferase (ALT). To measure the Serum lysozyme (LYZ) content, a commercial ELISA kit (Shanghai Enzyme-linked Biotechnology Co., Ltd., China) was utilized and followed the manufacturer's instructions.

### Antioxidant enzyme activities analysis

Antioxidant enzyme activities were measured according to the methods mostly followed in our lab (Laboratory of Fish Seed Engineering and Healthy Farming)^88,89,91^. Briefly, liver and head kidney samples were homogenized in nine (9) volumes of normal saline (Sodium Chloride Injection) and centrifuged at 7000 rpm for 10 min at 4 °C. The supernatants were collected for the following assays. Using commercial kits purchased from Nanjing Jiancheng Bioengineering Institute, Nanjing, Jiangsu, China (http://www.njjcbio.com), we examined the liver and head kidney of juvenile cobia fish for the presence of malondialdehyde (MDA), as well as superoxide dismutase (SOD), catalase (CAT), and glutathione peroxidase (GSH-PX). The manufacturer's protocol was followed for all kits. The SOD activity was measured by measuring the amount of enzyme necessary to inhibit 50% of ferricytochrome c reduction at 550 nm or units per mg of protein (U/mg + protein). GPX activity was expressed as units of GPX per mg soluble protein (U/mg · protein), with each unit defined as the amount of enzyme required to decrease the concentration of GSH in the reagent by 1 mol/L within a minute, after excluding non-enzymatic effects, and measured at 412 nm. A unit of CAT activity is defined as the amount of enzyme necessary to consume 1 mol H_2_O_2_ during 1 s at 37 °C, measured at 405 nm, and expressed as units per mg protein (U/mg · protein).

### Gut digestive enzyme activities analysis

We randomly selected five additional fish from each group to measure the intestinal digestive enzyme activity. Dissection of the fish took place after being euthanized, as previously described. After rinsing the fish's body with sterile distilled water and 70% ethanol, the dissection was performed using flame-sterilized scissors. Furthermore, to remove mucus from the intestine samples, they were washed with cold deionized water before being ground in a ratio of 1:9 (m/v) with cold sodium phosphate buffer (0.1 M, pH 7.0, 4 °C) as described previously by Yang et al.^92^ and Amenyogbe et al.^88^. The midgut homogenate was then centrifuged at 4 °C for 10 min at 3000×*g*. The supernatant containing enzymes was then stored at 80 °C until used. Assays were performed using the following kits obtained from Nanjing Jiancheng, Bioengineering Institute, China: Total protein quantitative kit, lipase kit, trypsin kit, and amylase kit. We analyzed total protein, amylase, trypsin, and lipase according to the manufacturer's instructions. The protein concentration was utilized to normalize the enzymatic activity.

### Challenge test after feeding trial

Eight fish from each tank were collected following the feeding trial, and each group consisted of three replicates. Injections were administered intraperitoneally with the pathogenic microbes (*Vibrio* *harveyi*) (acquired from the Provincial Key Laboratory of Pathogenic Biology and Epidemiology for Aquatic Animals, College of Fisheries, Guangdong Ocean University, Huguang Yan East, Zhanjiang 524088, Guangdong, China). The microorganisms particles were suspended, then the fish were injected 10^8^ CFU/mL 200 μL intra-peritoneally into the fish body dosage 200 μL/fish^2,88,89^. For 14 days, the combined survival of the fish was documented every 24 h to evaluate the impact of native isolates on the cobia resistance subsequently the injection of the pathogenic microorganisms.

### Gut gene expression

Using the method generally followed in our laboratory^88,89,91,93,94^, we used the Transgen Biotech TransZol UP Plus RNA kit (Beijing, China) to extract the total RNA from the midgut of juvenile cobias. We then dissolved the RNA in 120 μL of RNase-free water. An agarose gel stained with EB (ethidium bromide) was utilized to determine the quality of extracted RNA. The quality of extracted RNA was assessed using NanoDrop 2000 (Thermo Fisher Scientific). Quantitative reverse transcription PCR (RT-qPCR) was performed using Roche LightCycler^®^96 SW1.1 with TranScript cDNA Synthesis SuperMix (Transgen Biotech, Beijing, China) kit for quantitative cDNA synthesis. Table 6 lists the primer sequences utilized. Genomic data were screened from the cobias constructed in our laboratory. The primers were designed using Primer Premier 5.0 (https://primer-premier-5.software.informer.com) after verification by Blast (https://blast.ncbi.nlm.nih.gov/Blast.cgi)s (Table 6. Primer design was based on other species. Following are the names and GenBank accession numbers of the species used; *Anguilla* *anguilla* colony stimulating factor 1 receptor (XM_035409185.1); *Ctenopharyngodon* *idella* colony stimulating factor 1 receptor (KP244336.1); *Danio* *rerio* colony stimulating factor 1 receptor (BC162803.1); *Pygocentrus* *nattereri* colony stimulating factor 1 receptor (XM_017703791.2); *Epinephelus* *akaara* immunoglobulin (HQ007252.2); *Oncorhynchus* *mykiss* IgM (S63348.1); *Rachycentron* *canadum* immunoglobulin (JX025102.1); *Sparus* *aurata* immunoglobulin (JQ811851.4); *Takifugu* *rubripes* IgM (AB217624.1); *Epinephelus* *coioides* MHC class II (GU992883.1); *Oreochromis* *niloticus* MHC class II (MG882391.1); *Trachinotus* *ovatus* MHC class II (KU167010.1); *Epinephelus* *coioides* MHC class I (FJ896112.3); *Monopterus* *albus* MHC class I (KF468819.1); *Salmo* *salar* MHC class I (L26460.1); *Odontobutis* *potamophila* MHC class I (KP973945.1); *Dicentrarchus* *labrax* partial mRNA for T cell receptor beta (FN667952.1); *Lutjanus* *peru* T cell receptor beta (MF347708.1) and *Sparus* *aurata* mRNA for T-cell receptor beta (AM490438.1). Sequences were retrieved from GenBank and aligned using DNAman8 software. In a 20-μL PCR reaction, 10 μL of Transtart Tip Green qPCR Supermix (TransGen Biotech, China), 0.5 μL of each primer, 8 μL enzyme-free water, and 0.5 μL cDNA were used. To normalize gene expression, the β-actin gene was used as an internal reference. Using the melting curve, primer specificity was determined. The reaction conditions of RT-qPCR are as follows: pre-denaturation at 94 °C for 30 s; 40 cycles of denaturation at 94 °C for 5 s, annealing at 60 °C for 30 s and extension at 72 °C for 10 s; and final extension at 72 °C for 5 min^88,89^. In each case, triplicate samples were used to verify the results. The calculations were performed using the method of 2^−ΔΔCt^^95^.

### Extraction of genomic DNA and PCR amplifications

Total genomic DNA was extracted using the cetyltrimethylammonium bromide (CTAB) and sodium dodecyl sulfate (SDS) (CTAB/SDS) method. Deoxyribonucleic acid (DNA) concentration and purity were determined using electrophoresis in 1% agarose gels, and DNA samples were diluted to 1 ng/μL in sterile water. All Polymerase chain reaction (PCR) amplifications were carried out in a 15-μL final volume using Phusion^®^ High-Fidelity PCR Master Mix (New England Biolabs, Ipswich, MA, USA), 0.2 μM of forward and reverse primers, and 10 ng of template DNA. Thermal cycling conditions consisted of initial denaturation at 98 °C for 1 min, followed by 30 cycles of denaturation at 98 °C for 10 s, annealing at 50 °C for 30 s, and elongation at 72 °C for 30 s, with a final step at 72 °C for 5 min. PCR products were purified using Qiagen Gel Extraction Kit (Qiagen, Germany).

### Library preparation and shotgun metagenomic sequencing

Sequencing libraries were generated using TruSeq^®^ DNA PCR-Free Sample Preparation Kit (Illumina, San Diego, CA, USA) following the manufacturer's recommendations and index codes were added. Library quality was assessed in Qubit@ 2.0 Fluorometer (Thermo Scientific, Waltham, MA, USA) and Agilent Bioanalyzer 2100 system (Agilent Technologies, CA, USA). Library was sequenced in Illumina HiSeq 2000 sequencing system, and 250 bp paired-end reads were generated^96^.

### Sequencing data analysis

Paired-end reads were assigned to samples based on specific barcodes and truncated by eliminating barcode and primer sequence. Paired-end reads were merged using FLASH v.1.2.7 (http://ccb.jhu.edu/software/FLASH/)^97^, FLASH is a fast and accurate analysis tool designed to merge paired-end reads in which at least some of the reads overlap the read generated from the opposite end of the same DNA fragment. Splicing sequences were called raw tags. Quality filtering on raw tags was performed under specific filtering conditions to obtain high-quality clean tags^98^ using QIIME v.1.9.1 (http://qiime.org/scripts/split_libraries_fastq.html)^99^ for quality control. Tags were compared with sequences in the Silva database (https://www.arb-silva.de/) using the UCHIME algorithm (UCHIME Algorithm, http://www.drive5.com/usearch/manual/uchime_algo.html)^100^ to detect chimera sequences which were removed^101^, therefore yielding effective tags.

### Operational taxonomic unit (OTU) clustering and species annotation

Operational taxonomic units (OTUs) clusters were generated by UPARSE software v.7.0.1001 (http://drive5.com/uparse/)^102^. Sequences with ≥ 97% similarity were assigned to the same OTUs. A representative sequence for each OTU was screened for further annotation. The Silva Database (http://www.arb-silva.de/)^103^ was used for each representative sequence based on the Mothur algorithm to annotate taxonomic information. To evaluate the phylogenetic relationship of different OTUs and differences in the dominant species in different samples groups, multiple sequence alignment was conducted using MUSCLE software v.3.8.31 (http://www.drive5.com/muscle/)^104^. OTUs abundance was normalised using a standard sequence number corresponding to the sample with the lowest number of sequences. Subsequent analyses of alpha and beta diversity were performed based on normalised data^105^.

### Metabolite extraction and metabolic profiling

Six intestinal contents samples from both treated and control groups were thawed on ice, and 50 ± 2 mg of each sample were taken, which were mixed with cold steel beads and submitted to homogenization at 30 Hz for 3 min. Then, 1 mL of 70% methanol as an internal standard extract was added to the homogenised centrifuge tubes, and the mixture was vortexed for 5 min and submitted to centrifugation at 12,000 rpm at 4 °C for 10 min. After centrifugation, 400 µL of supernatant was transferred to the corresponding EP tube and stored at − 20 °C overnight. Subsequently, tubes were centrifuged at 12,000 rpm at 4 °C for 3 min, and 2 mL of the supernatant was taken in the liner of the corresponding injection bottle for on-board analysis.

For T3 Ultra-Performance Liquid Chromatography (UPLC), sample extracts were analysed using an ExionLC™ QTRAP^®^ AD LC–ESI–MS/MS system (Sciex, Framingham, MA, USA; https://sciex.com.cn/). Analytical conditions were as follows: UPLC column, Waters ACQUITY UPLC HSS T3 C18 (1.8 µm, 2.1 mm × 100 mm); column temperature, 40 °C; flow rate, 0.4 mL/min; injection volume, 2 μL; solvent system, water (0.1% formic acid): acetonitrile (0.1% formic acid); gradient program, 95:5 V/V at 0 min, 10:90 V/V at 11 min, 10:90 V/V at 12 min, 95: 5 V/V at 12.10 min, 95: 5 V/V at 14 min.

For amide UPLC conditions, sample extracts were analysed using ExionLC™ AD QTRAP^®^ LC–ESI–MS/MS system (Sciex). Analytical conditions were as follows: UPLC column, Waters ACQUITY UPLC BEH Amide 1.7 µm, 2.1 mm × 100 mm; column temperature, 40 °C; flow rate, 0.4 mL/min; injection volume, 2 μL; solvent system, water (25 mM ammonium formate/0.4% ammonia): acetonitrile; gradient program, 10:90 V/V at 0 min, 40:60 V/V at 9.0 min, 60:40 V/V at 10 min, 60:40 V /V at 11 min, 10:90 V/V at 11.10 min, 10:90 V/V at 15 min. LIT and triple quadrupole (QQQ) scans were acquired on a triple quadrupole-linear ion trap mass spectrometer (QTRAP^®^) LC–MS/MS System equipped with an ESI turbo ion-spray interface operating in positive and negative ion mode and controlled by Analyst software v.1.6.3 (Sciex). ESI source operation parameters were as follows: source temperature, 500 °C; ion spray voltage (IS), 5500 V (positive) and − 4500 V (negative); ion source gas I (GSI), gas II (GSII), and curtain gas (CUR) were set at 55, 60, and 25.0 psi, respectively; collision gas (CAD) was high. Instrument and mass calibration was performed with polypropylene glycol solutions at 10 and 100 μmol/L in QQQ and LIT modes respectively. A specific set of MRM transitions was monitored for each period according to the metabolites eluted within the referred period^106^.

### Data analysis

The data for growth parameters, biochemistry, enzymes activities, challenge test, were analyzed using SPSS (IBM SPSS STATISTICS, 16.0 package, IBM Corporation, New York, United States) for Windows version 7.0 (SPSS, Chicago, United States). An Independent t‑test was utilized to compare the variable between the two groups. The data were expressed as mean ± standard error (SE). *P* < 0.05 was considered statistically significant. Different letters were used to indicate the statistically significant differences. The methods described by Xia et al.^107^ were adopted with slight modifications. In brief, clusters of metabolomic data were examined by principal component analysis (PCA). The possible outliers were identified with a 95% confidence interval (CI) threshold. Furthermore, the hierarchical clustering algorithm was employed to generate heat maps to visualize the metabolite heterogeneity in the dataset. Subsequently, the intestinal microbiome profiles between samples and control were compared using principal coordinate analysis (PCoA). The heterogeneous intestinal microbiota compositions were evaluated through nonparametric test using Metastats (http://metastats.cbcb.umd.edu/) according to a previous report^107^ (White et al. 2009). Species analysis with significant differences (*P* < 0.05) between groups was conducted with R software using the functions intergroup T_test tests and map.

Hierarchical cluster analysis (HCA) data are presented as heatmaps with dendrograms, while Pearson correlation coefficients (PCC) were calculated using the cor function in R and presented as only heatmaps. Both HCA and PCC were carried out in R package ComplexHeatmap. For HCA, normalized signal intensities of metabolites (unit variance scaling) are visualized as a colour spectrum. Differential metabolites were identified based on VIP ≥ 1 and absolute log_2_FC ≥ 1. VIP values were extracted from orthogonal partial least-squares discriminant analysis (OPLS-DA) which comprises score plots and permutation plots generated using the MetaboAnalystR package. Data was converted in log_2_ and mean centred before conducting orthogonal projections to latent structures-discriminant analysis. In order to avoid overfitting, a permutation test (200 permutations) was performed.

Identified metabolites were annotated using the KEGG Compound database (http://www.kegg.jp/kegg/compound/), and annotated metabolites were then mapped to the KEGG Pathway database (http://www.kegg.jp/kegg/pathway.html). Significantly enriched pathways are identified with a hypergeometric test's P-value for a given list of metabolites. Finally, Pearson's correlation coefficient was adopted to generate the correlation matrix between gut microbial species and associated metabolites.

### Statistical analysis

The data were analyzed using SPSS (IBM SPSS STATISTICS, 16.0 package, IBM Corporation, New York, United States) for Windows version 7.0 (SPSS, Chicago, United States). An Independent t‑test was utilized to compare the variable between the two groups. The data were expressed as mean ± standard error (SE). *P* < 0.05 was considered statistically significant. Different letters were used to indicate the statistically significant differences. The methods described by Xia et al.^107^ were adopted with slight modifications. In brief, clusters of metabolomic data were examined by principal component analysis (PCA). The possible outliers were identified with a 95% confidence interval (CI) threshold. Furthermore, the hierarchical clustering algorithm was employed to generate heat maps to visualize the metabolite heterogeneity in the dataset. Subsequently, the intestinal microbiome profiles between samples and control were compared using principal coordinate analysis (PCoA). The heterogeneous intestinal microbiota compositions were evaluated through nonparametric test using Metastats (http://metastats.cbcb.umd.edu/) according to a previous report^107^. Species analysis with significant differences (*P* < 0.05) between groups was conducted with R software using the functions intergroup T_test tests and map.

Hierarchical cluster analysis (HCA) data are presented as heatmaps with dendrograms, while Pearson correlation coefficients (PCC) were calculated using the cor function in R and presented as only heatmaps. Both HCA and PCC were carried out in R package ComplexHeatmap. For HCA, normalized signal intensities of metabolites (unit variance scaling) are visualized as a colour spectrum. Differential metabolites were identified based on VIP ≥ 1 and absolute log_2_FC ≥ 1. VIP values were extracted from orthogonal partial least-squares discriminant analysis (OPLS-DA) which comprises score plots and permutation plots generated using the MetaboAnalystR package. Data was converted in log_2_ and mean centred before conducting orthogonal projections to latent structures-discriminant analysis. In order to avoid overfitting, a permutation test (200 permutations) was performed.

Identified metabolites were annotated using the KEGG Compound database (http://www.kegg.jp/kegg/compound/), and annotated metabolites were then mapped to the KEGG Pathway database (http://www.kegg.jp/kegg/pathway.html). Significantly enriched pathways are identified with a hypergeometric test's P-value for a given list of metabolites. Finally, Pearson's correlation coefficient was adopted to generate the correlation matrix between gut microbial species and associated metabolites.

### Ethics statement

The Guangdong Ocean University Research Council approved this animal research (approval number: GDOU-LAE-2020-013). Also followed the recommendations in the ARRIVE guidelines. All methods were carried out in accordance with relevant guidelines and regulations.

## Supplementary Information

Supplementary Figure 1.

Supplementary Figure 2.

Supplementary Figure 3.

Supplementary Figure 4.

Supplementary Figure 5.

Supplementary Legends.

Supplementary Table 1.

## Data Availability

Reads were deposited on the National Centre for Biotechnology Information (NCBI) database under the NCBI Bio-project PRJNA786050. The datasets generated and/or analysed during the current study are available in the [NCBI] repository, [https://www.ncbi.nlm.nih.gov/biosample; include the BioSample accessions SAMN23614717, SAMN23614718, SAMN23614719, SAMN23614720, SAMN23614721, SAMN23614722, SAMN23614723, SAMN23614724, SAMN23614725, SAMN23614726, SAMN23614727 and SAMN23614728]” Other data supporting the results of this study are obtainable from the corresponding author (Prof. Chen Gang, email: cheng@gdou.edu.cn) upon reasonable demand.
