# Supplementary figures and images for: Effects of autochthonous strains mixture on gut microbiota and metabolic profile in cobia (Rachycentron canadum)
Source: Sci Rep. 2022 Oct 18;12:17410. doi: 10.1038/s41598-022-19663-x (PMC9579153; doi:10.1038/s41598-022-19663-x)

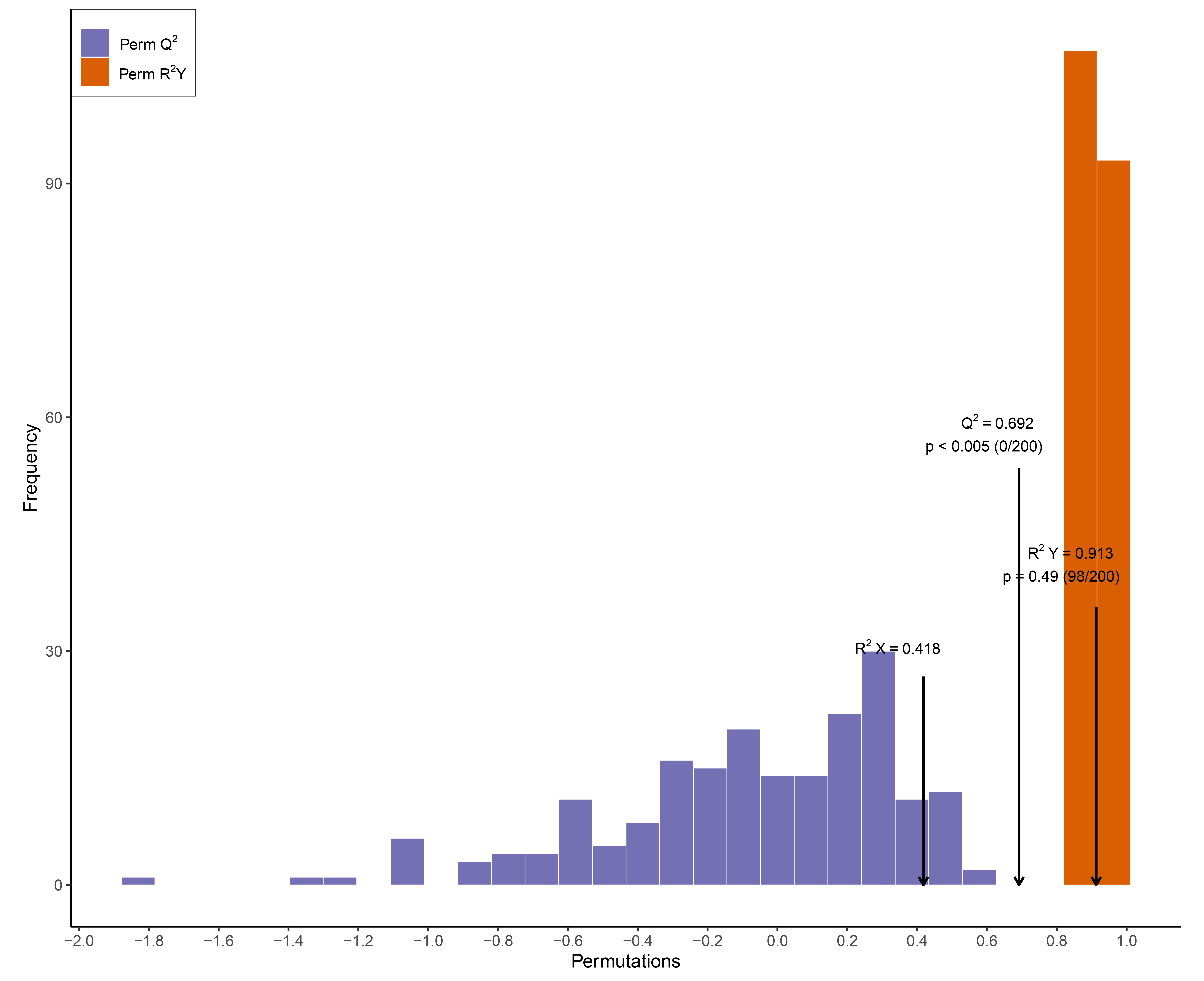

Supplement: Supplementary file 1 — Supplementary Figure 1. [file 41598_2022_19663_MOESM1_ESM.tif]

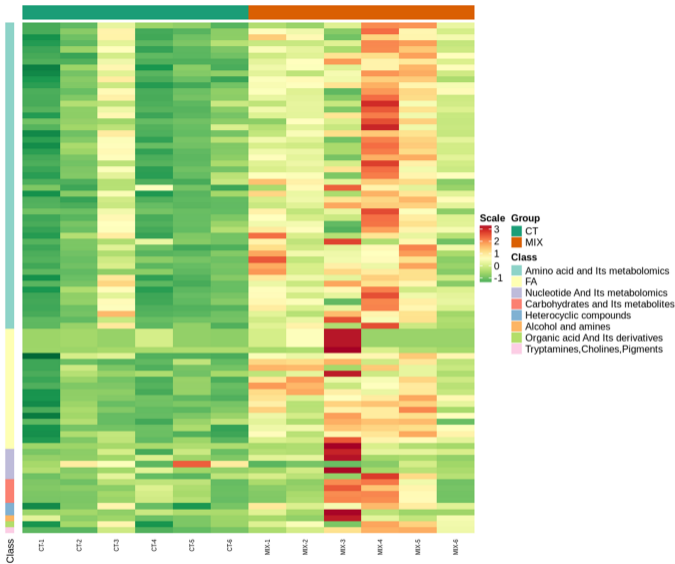

2A

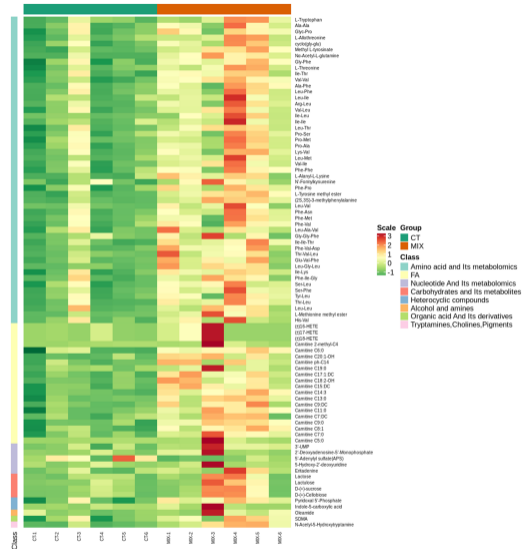

2B

Supplement: Supplementary file 2 — Supplementary Figure 2. [file 41598_2022_19663_MOESM2_ESM.pdf]

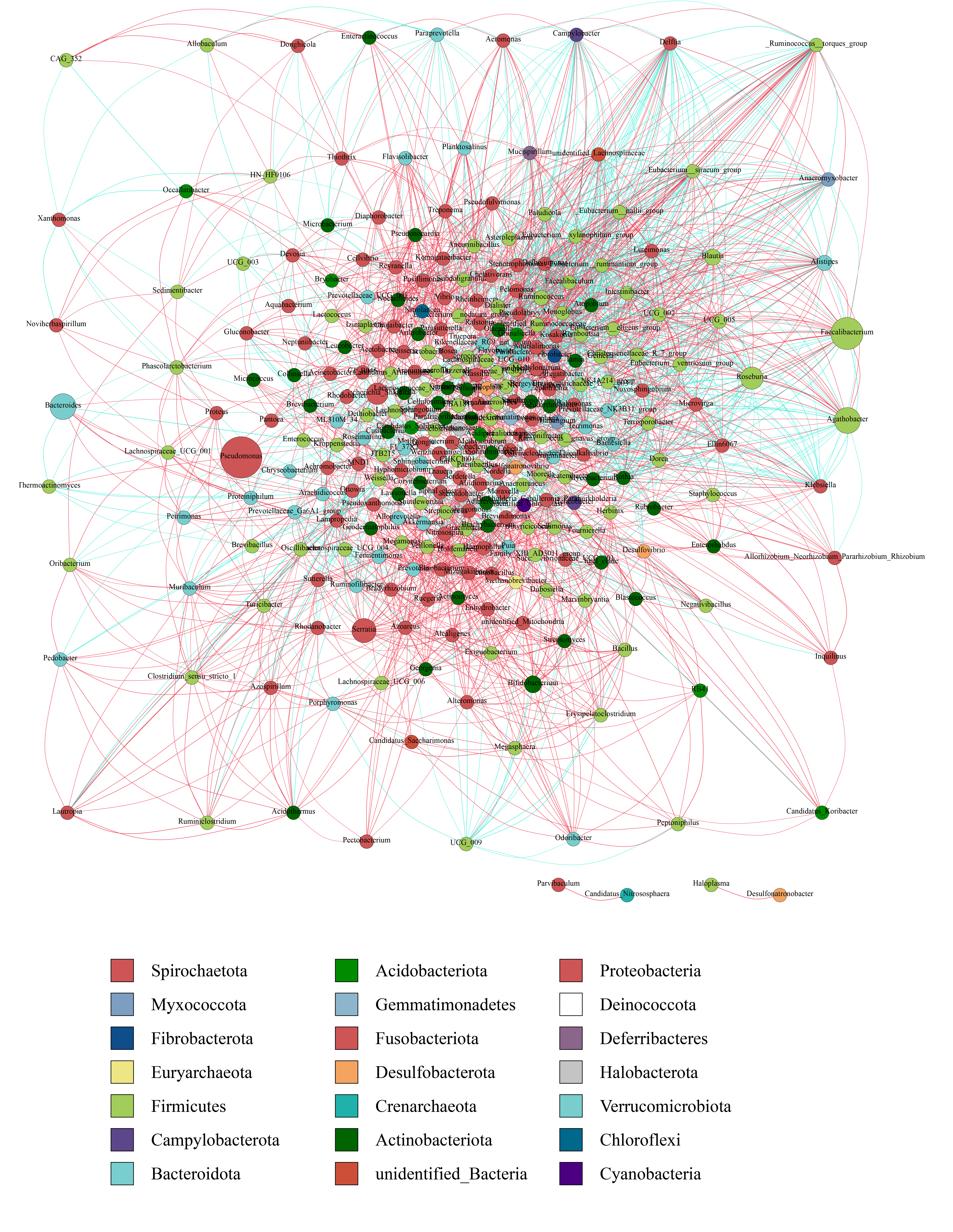

Supplement: Supplementary file 3 — Supplementary Figure 3. [file 41598_2022_19663_MOESM3_ESM.tif]

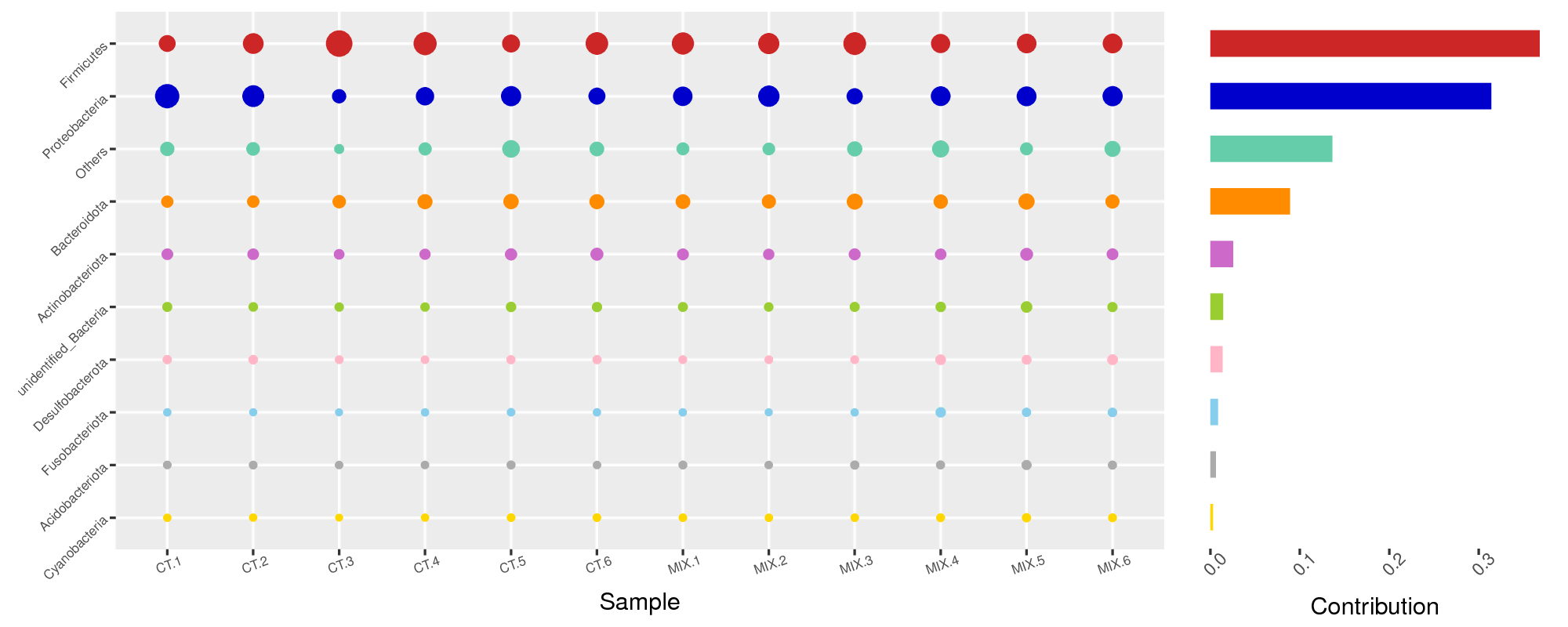

Supplement: Supplementary file 5 — Supplementary Figure 5. [file 41598_2022_19663_MOESM5_ESM.tiff]
