# Supplementary material for: Effects of autochthonous strains mixture on gut microbiota and metabolic profile in cobia (Rachycentron canadum)
Source: Sci Rep. 2022 Oct 18;12:17410. doi: 10.1038/s41598-022-19663-x (PMC9579153; doi:10.1038/s41598-022-19663-x)
Supplement: Supplementary file 6 — Supplementary Legends. [file 41598_2022_19663_MOESM6_ESM.docx]

**Supplementary Figure 1.** The OPLS-DA model for metabolic change examination was effective, in which R2X = 0.418, R2Y = 0.913, and O2 = 0.692.

**Supplementary Figure 2A and 2B.** **Clustering analysis of differential metabolites of KEGG signaling pathways**. Note: Horizontal is sample information, longitudinal is metabolite information, Scale is the value obtained after standardizing the relative content of metabolites (the color is more red, the higher the content), Group is grouped, and Class is the metabolite classification. Among them, the all_heatmap_class: heat map classified by metabolites; all_heatmap_col-row_cluster: cluster analysis of metabolites and samples, the cluster line on the left side of the figure is the metabolite cluster line, and the cluster line in the upper part of the figure is the sample cluster line; all_heatmap_row_cluster: only the metabolites are clustered, and the cluster line on the left side of the figure is the metabolite cluster line. C: control group; MIX: autochthonous strains supplementation group.

**Supplementary Figure 3.** Inter-omic Pearson correlation networks. Note: The specific pair-wise Pearson correlation was determined for every pair of each operational taxonomic unit (OTU) and metabolite. The circles represent OTU, and different colours indicate different classifications (level on phylum).

**Supplementary Figure 4. Pearson correlation hierarchical cluster heat map of differential microorganisms and differential metabolites. Note:** Rows represent microorganisms and columns represent metabolites. The evolutionary tree on the left represents the microbial-level clustering results, and the evolutionary tree above represents the hierarchical clustering results of metabolites. Red indicates positive correlation and blue indicates negative correlation. The p-value of the significance test for the correlation coefficients is significantly different < 0.05, denoted by "*", and the p-value < 0.01 is significantly different, denoted by "**". 7A and 7B are different levels of classification of microorganisms based on Phylum and Genus.

**Supplementary Figure 5.** Simper Difference Contribution. Note: The top 10 contributing species are selected for mapping by default. The vertical axis represents the species, the horizontal axis is the sample, the bubble size represents the relative abundance of the species, and the Contribution is the contribution of the species to the differences between the two groups.
