# Supplementary material for: Effects of autochthonous strains mixture on gut microbiota and metabolic profile in cobia (Rachycentron canadum)
Source: Sci Rep. 2022 Oct 18;12:17410. doi: 10.1038/s41598-022-19663-x (PMC9579153; doi:10.1038/s41598-022-19663-x)
Supplement: Supplementary file 7 — Supplementary Table 1. [file 41598_2022_19663_MOESM7_ESM.docx]

**Supplementary Table 1 The composition of the 85 known differential metabolites.**

| #ID | Name | VIP | p_value | Fold_Change | Log2FC | regulated |
| --- | --- | --- | --- | --- | --- | --- |
| MADN0006 | L-Tryptophan | 1.659463825 | 0.035953899 | 2.498878903 | 1.32128099 | up |
| MADN0012 | Ala-Ala | 1.450165965 | 0.034293709 | 2.444535842 | 1.289560559 | up |
| MADN0013 | Glyc-Pro | 1.392485946 | 0.016942996 | 2.336239316 | 1.224188067 | up |
| MADN0056 | Lactose | 1.400269265 | 0.134502284 | 2.450664826 | 1.293173182 | up |
| MADN0057 | Lactulose | 1.295656195 | 0.142296597 | 2.439533684 | 1.286605404 | up |
| MADN0181 | D-(+)-sucrose | 1.347927925 | 0.125319997 | 2.541718264 | 1.345804124 | up |
| MADN0275 | Pyridoxal 5'-Phosphate | 1.754690364 | 0.005920937 | 2.130634785 | 1.09128332 | up |
| MADN0343 | 3'-UMP | 1.628786694 | 0.081377834 | 2.073336232 | 1.051954096 | up |
| MADN0370 | D-(+)-Cellobiose | 1.376049344 | 0.118314992 | 2.396207492 | 1.260752839 | up |
| MADN0388 | L-Allothreonine | 1.629214996 | 0.022568644 | 2.063450682 | 1.045058957 | up |
| MADN0467 | cyclo(gly-glu) | 1.545802277 | 0.017969525 | 2.549795381 | 1.350381477 | up |
| MADP0054 | 2'-Deoxyadenosine-5'-Monophosphate | 1.534359431 | 0.20785312 | 2.951166651 | 1.561285392 | up |
| MADP0115 | Methyl L-tyrosinate | 1.945189089 | 0.000621209 | 4.269367875 | 2.09402248 | up |
| MADP0130 | Nα-Acetyl-L-glutamine | 2.016277716 | 0.000937442 | 7.470228045 | 2.901152285 | up |
| MADP0131 | Gly-Phe | 1.653281304 | 0.007343492 | 2.04823001 | 1.034377734 | up |
| MADP0135 | L-Threonine | 1.711296477 | 0.008415084 | 2.032610719 | 1.02333394 | up |
| MADP0187 | Ile-Thr | 1.812055493 | 0.009559746 | 2.375124204 | 1.248002959 | up |
| MADP0211 | Val-Val | 1.948623533 | 8.37467E-05 | 2.643829742 | 1.402629273 | up |
| MADP0332 | Ala-Phe | 1.240354528 | 0.029445358 | 3.155546076 | 1.657889689 | up |
| MADP0339 | Leu-Phe | 1.401785458 | 0.050789121 | 2.30121006 | 1.202392683 | up |
| MADP0373 | Leu-Ile | 1.434210804 | 0.047544985 | 2.275475228 | 1.18616788 | up |
| MADP0376 | Arg-Leu | 1.341655265 | 0.031039434 | 4.323738572 | 2.112279295 | up |
| MADP0384 | Val-Leu | 1.750333262 | 0.011826482 | 2.42735039 | 1.279382378 | up |
| MADP0392 | Ile-Leu | 1.675917243 | 0.029379298 | 2.725075866 | 1.446296395 | up |
| MADP0393 | Ile-Ile | 1.395159273 | 0.070269274 | 2.205027454 | 1.140796618 | up |
| MADP0397 | Leu-Thr | 1.745254048 | 0.011151039 | 2.221383091 | 1.151458216 | up |
| MADP0399 | Pro-Ser | 1.237543352 | 0.038420445 | 2.353839084 | 1.235015697 | up |
| MADP0403 | Pro-Met | 1.001287195 | 0.043735725 | 2.239452933 | 1.163146345 | up |
| MADP0404 | Pro-Ala | 1.502039718 | 0.021384062 | 2.460516275 | 1.298961059 | up |
| MADP0410 | Lys-Val | 1.436462076 | 0.023569927 | 2.531867359 | 1.340201826 | up |
| MADP0415 | Leu-Met | 1.769046319 | 0.013247355 | 3.54900997 | 1.827416627 | up |
| MADP0429 | Val-Ile | 1.761904049 | 0.009511004 | 2.533620206 | 1.341200278 | up |
| MEDN0624 | 5'-Adenylyl sulfate(APS) | 1.324725155 | 0.055760934 | 0.395560924 | -1.33802818 | down |
| MEDN0753 | (±)16-HETE | 1.282648674 | 0.226411807 | 3.464523786 | 1.792657061 | up |
| MEDN0755 | (±)17-HETE | 1.282648674 | 0.226411807 | 3.464523786 | 1.792657061 | up |
| MEDN0757 | (±)18-HETE | 1.282648674 | 0.226411807 | 3.464523786 | 1.792657061 | up |
| MEDN1513 | 5-Hydroxy-2'-deoxyuridine | 1.277384027 | 0.312045558 | 2.216902511 | 1.148545329 | up |
| MEDP0078 | Phe-Phe | 1.13855523 | 0.012953604 | 3.124752196 | 1.643741784 | up |
| MEDP0087 | L-Alanyl-L-Lysine | 1.781990971 | 0.003862017 | 2.513392738 | 1.329636122 | up |
| MEDP0212 | N-Acetyl-5-Hydroxytryptamine | 1.938136737 | 0.004261598 | 3.829774973 | 1.937259626 | up |
| MEDP0326 | N'-Formylkynurenine | 1.427646721 | 0.051630812 | 2.806932134 | 1.488994183 | up |
| MEDP0574 | Oleamide | 1.306751155 | 0.146458456 | 2.656801529 | 1.409690458 | up |
| MEDP0618 | Carnitine 2-methyl-C4 | 1.400276738 | 0.277474378 | 3.670097044 | 1.875818211 | up |
| MEDP0637 | Phe-Pro | 1.517064417 | 0.007270173 | 2.416255807 | 1.2727732 | up |
| MEDP0664 | L-Tyrosine methyl ester | 2.014513474 | 0.000165097 | 3.807406949 | 1.928808778 | up |
| MEDP1063 | Indole-5-carboxylic acid | 1.163851179 | 0.294642899 | 2.210529427 | 1.14439194 | up |
| MEDP1289 | Carnitine C6:0 | 1.64210384 | 0.003684272 | 2.062065084 | 1.044089869 | up |
| MEDP1307 | (2S,3S)-3-methylphenylalanine | 2.090353634 | 0.001411753 | 4.007429519 | 2.002677147 | up |
| MEDP1377 | Carnitine C20:1-OH | 1.983128021 | 0.001159948 | 2.764856201 | 1.467204449 | up |
| MEDP1382 | Carnitine ph-C14 | 1.612850076 | 0.006529499 | 3.176050978 | 1.667234069 | up |
| MEDP1384 | Carnitine C19:0 | 1.653014758 | 0.084929396 | 2.374140243 | 1.247405159 | up |
| MEDP1385 | Carnitine C17:1:DC | 1.944310928 | 0.000588339 | 2.809973835 | 1.490556697 | up |
| MEDP1386 | Carnitine C18:2-OH | 1.903392338 | 0.004099221 | 3.493859108 | 1.804821432 | up |
| MEDP1394 | Carnitine C15:DC | 1.936119663 | 0.000375924 | 2.678165195 | 1.421244952 | up |
| MEDP1408 | Carnitine C14:3 | 1.952241862 | 5.94659E-05 | 3.164671534 | 1.662055768 | up |
| MEDP1411 | Carnitine C13:0 | 1.855476363 | 0.000573692 | 2.532431479 | 1.340523234 | up |
| MEDP1415 | Carnitine C9:DC | 1.874897869 | 0.006985524 | 3.70579151 | 1.889781717 | up |
| MEDP1416 | Carnitine C11:0 | 1.678112651 | 0.000355732 | 2.557514155 | 1.354742225 | up |
| MEDP1423 | Carnitine C7:DC | 1.493936391 | 0.032857635 | 2.082667124 | 1.05843227 | up |
| MEDP1424 | Carnitine C9:0 | 2.013080618 | 2.52476E-05 | 2.607795323 | 1.382830642 | up |
| MEDP1428 | Carnitine C8:1 | 1.73435562 | 0.000646109 | 3.116911592 | 1.640117235 | up |
| MEDP1430 | Carnitine C7:0 | 1.984893095 | 0.005791145 | 2.222344829 | 1.152082689 | up |
| MEDP1440 | Carnitine C5:0 | 1.400276738 | 0.277474378 | 3.670097044 | 1.875818211 | up |
| MEDP1488 | SDMA | 1.066038539 | 0.035144872 | 2.434428729 | 1.283583265 | up |
| MEDP1506 | Leu-Val | 1.824768982 | 0.038566554 | 2.937777983 | 1.554725371 | up |
| MEDP1507 | Phe-Asn | 1.739518824 | 0.01186336 | 3.071770889 | 1.619070615 | up |
| MEDP1508 | Phe-Met | 1.240729937 | 0.038995868 | 2.678450906 | 1.421398853 | up |
| MEDP1512 | Phe-Val | 1.143523306 | 0.027131611 | 2.744756933 | 1.456678394 | up |
| MEDP1668 | Leu-Ala-Val | 1.429329475 | 0.053501443 | 2.111321031 | 1.07814596 | up |
| MEDP1670 | Gly-Gly-Phe | 1.614174334 | 0.100088693 | 2.09104732 | 1.06422571 | up |
| MEDP1673 | Ile-Ile-Thr | 1.884946364 | 0.001762086 | 3.751290927 | 1.907387154 | up |
| MEDP1676 | Phe-Val-Asp | 1.895216819 | 0.004029181 | 5.233493346 | 2.387774264 | up |
| MEDP1678 | Thr-Val-Leu | 1.874378379 | 0.023379321 | 3.683243369 | 1.880976726 | up |
| MEDP1679 | Glu-Val-Phe | 1.850477849 | 0.007878348 | 3.77277638 | 1.915626591 | up |
| MEDP1681 | Leu-Gly-Leu | 2.050737344 | 0.006914737 | 6.836956935 | 2.773354338 | up |
| MEDP1870 | Ile-Lys | 1.734954938 | 0.010015444 | 2.233368841 | 1.159221532 | up |
| MEDP1881 | Phe-Ile-Gly | 1.77675877 | 0.047549135 | 2.652936802 | 1.407590308 | up |
| MEDP1888 | Ser-Leu | 1.694580824 | 0.010138327 | 2.132099064 | 1.092274472 | up |
| MEDP1889 | Ser-Phe | 1.727786013 | 0.008796746 | 2.970780741 | 1.570842131 | up |
| MEDP1894 | Tyr-Leu | 1.233764395 | 0.030088032 | 2.847638433 | 1.509765978 | up |
| MEDP1912 | Thr-Leu | 1.892656562 | 0.003844986 | 2.259651773 | 1.176100461 | up |
| MEDP1937 | Leu-Leu | 1.754553817 | 0.075042791 | 2.047908075 | 1.034150958 | up |
| MEDP1953 | Eritadenine | 1.272786463 | 0.097421245 | 2.749514109 | 1.45917669 | up |
| MEDP1970 | L-Methionine methyl ester | 2.094766519 | 0.013348476 | 4.195985819 | 2.069009802 | up |
| MEDP1987 | His-Val | 1.506908372 | 0.093517415 | 2.07876147 | 1.055724224 | up |
